# Dissociating error-based and reinforcement-based loss functions during sensorimotor learning

**DOI:** 10.1371/journal.pcbi.1005623

**Published:** 2017-07-28

**Authors:** Joshua G. A. Cashaback, Heather R. McGregor, Ayman Mohatarem, Paul L. Gribble

**Affiliations:** 1 Brain and Mind Institute, Department of Psychology, Western University, London, ON, Canada; 2 Graduate Program in Neuroscience, Western University, London, ON, Canada; 3 Department of Biology, Western University, London, ON, Canada; 4 Department of Physiology and Pharmacology, Western University, London, ON, Canada; Imperial College London, UNITED KINGDOM

## Abstract

It has been proposed that the sensorimotor system uses a loss (cost) function to evaluate potential movements in the presence of random noise. Here we test this idea in the context of both error-based and reinforcement-based learning. In a reaching task, we laterally shifted a cursor relative to true hand position using a skewed probability distribution. This skewed probability distribution had its mean and mode separated, allowing us to dissociate the optimal predictions of an error-based loss function (corresponding to the mean of the lateral shifts) and a reinforcement-based loss function (corresponding to the mode). We then examined how the sensorimotor system uses error feedback and reinforcement feedback, in isolation and combination, when deciding where to aim the hand during a reach. We found that participants compensated differently to the same skewed lateral shift distribution depending on the form of feedback they received. When provided with error feedback, participants compensated based on the mean of the skewed noise. When provided with reinforcement feedback, participants compensated based on the mode. Participants receiving both error and reinforcement feedback continued to compensate based on the mean while repeatedly missing the target, despite receiving auditory, visual and monetary reinforcement feedback that rewarded hitting the target. Our work shows that reinforcement-based and error-based learning are separable and can occur independently. Further, when error and reinforcement feedback are in conflict, the sensorimotor system heavily weights error feedback over reinforcement feedback.

## Author Summary

Whether serving a tennis ball on a gusty day or walking over an unpredictable surface, the human nervous system has a remarkable ability to account for uncertainty when performing goal-directed actions. Here we address how different types of feedback, error and reinforcement, are used to guide such behavior during sensorimotor learning. Using a task that dissociates the optimal predictions of error-based and reinforcement-based learning, we show that the human sensorimotor system uses two distinct loss functions when deciding where to aim the hand during a reach—one that minimizes error and another that maximizes success. Interestingly, when both of these forms of feedback are available our nervous system heavily weights error feedback over reinforcement feedback.

## Introduction

Error feedback and reinforcement feedback can each guide motor adaptation in a visuomotor rotation task [1]. It has been proposed that error feedback and reinforcement feedback engage different neural mechanisms [2]. Indeed, depending on the form of feedback used during adaptation, newly acquired motor commands differ in terms of generalizability [1] and retention [3]. For error-based learning, it is suggested that adaptation occurs by minimizing error to update an internal model [4]. For reinforcement-based learning, it is proposed that adaptation is model-free and occurs by sampling motor outputs to find a set that maximizes the probability of task success [5]. Importantly, both these forms of learning can occur in the presence of internally [6, 7] and externally [8, 9] generated random variability (noise).

Loss functions are central to several current computational theories of sensorimotor control [10]. An error-based loss function describes the relationship between potential movements and the associated costs of error [9]. In other words, an error-based loss function describes how errors of different magnitudes are penalized. A reinforcement-based loss function describes the relationship between potential movements and the associated probabilities of reward (or punishment) [7]. The idea that the sensorimotor system uses a loss function to select a statistically optimal movement in the presence of noise has been examined in tasks involving error feedback [9, 11], reinforcement feedback [1], and both error and reinforcement feedback together [1, 3, 7, 12].

In tasks involving error feedback, it has been proposed that the sensorimotor system may use an error-based loss function to select movements [9]. As an example, here we will describe how an absolute error loss function and a squared error loss function can be used to select aim location during a game of darts. In this context, error refers to the distance of an individual dart to the bullseye. Let us assume that, after several throws, when attempting to aim the darts at a particular location that there is some spread, or distribution, of darts on a board. If the chosen strategy is to minimize absolute error around the bulls-eye, one should adjust their aim until the sum of the absolute distances of the darts is at its lowest possible value. This strategy corresponds to selecting a single aim location that minimizes the cost (output) of an absolute error loss function (i.e., ∑|*error*_*i*_|^1^). This absolute error loss function linearly weights individual error magnitudes and would result in the median of the dart distribution being directly over the bullseye. Similarly, if the chosen strategy is to minimize squared error around the bullseye, one should adjust their aim such that the sum of the squared distances of the darts is at its lowest possible value. This strategy corresponds to selecting a single aim location that minimizes the cost of a squared error loss function (i.e., ∑|*error*_*i*_|^2^). The squared error loss function places a heavier emphasis on minimizing large errors relative to smaller errors, in a quadratic fashion, and would result in the mean of the dart distribution being directly over the bullseye.

Using tasks that involve noisy error feedback, some researchers have reported that sensorimotor behavior is best represented with an error-based loss function where the exponent on the error term is between 1 (absolute error) and 2 (squared error) [9, 13], while others report that an exponent of 2 best fits behavior [11, 14, 15]. Based on these works, it is possible that the error-based loss function that most aligns with behavior may to some extent be task dependent.

The concept of loss functions also extends to sensorimotor tasks involving reinforcement feedback, where the goal is maximize task success [5]. The optimal movement that maximizes the probability of success can be found by minimizing the 0-1 loss function [9, 16]. Again, we can describe this reinforcement-based loss function using a distribution of darts on a board. With the 0-1 loss function, every dart that hits the bulls-eye is assigned a value of 0 and each dart that misses the bulls-eye is assigned a value of 1. To minimize this loss function, one should adjust their aim such that the greatest number of darts hit the bulls-eye. This strategy maximizes the probability of success (by minimizing failure) and corresponds to placing the mode of the distribution of darts directly over the bullseye. This loss function can easily be extended to account for graded reinforcement feedback, where either the magnitude [7, 17] or probability [18] of reinforcement feedback varies according to some function that is externally imposed by the experimenter.

It has recently been shown that the sensorimotor system can maximize task success when using only binary reinforcement feedback. Shadmehr and colleagues used a visuomotor rotation task, where the true target was displaced from the displayed target by some small amount [1, 18]. In line with a reinforcement-based loss function and without any error feedback, they found that participants were able to adapt where they aimed their hand using only reinforcement feedback that signaled whether or not the true target had been hit.

Researchers have also explored how reinforcement feedback and error feedback are used when provided simultaneously [1, 3, 7, 12, 17, 19]. This has been done for a range of tasks and has yielded mixed results. In studies reported by Trommershäuser and colleagues [7; 20–23], participants performed rapid reaches with continuous visual feedback of their hand (visual error feedback) to a large rewarding target (positive reinforcement) with an overlapping punishment region (negative reinforcement). Participants learned to aim to a location that maximized reward. Conversely, others have provided evidence that continuous error feedback dominates over, or perhaps suppresses, reinforcement feedback during a visuomotor rotation task [3, 12, 24]. Izawa and Shadmehr (2011) suggested that with a decrease in error feedback quality, the sensorimotor system might increase its reliance on reinforcement feedback. However, a feature of these experiments is that they did not separate the predictions for where participants should aim their hand when receiving reinforcement feedback or error feedback. Such separation would provide a powerful way to investigate how the sensorimotor system weights the relative influence of error feedback and reinforcement feedback when they are provided in combination.

Here, we designed two experimental reaching tasks that separate the predictions of error-based and reinforcement-based loss functions on where to aim the hand. In doing so, we promoted dissociation in behavior simply by manipulating the form of feedback provided to participants. Participants reached to visual targets without vision of their arm. Unbeknownst to participants, visual feedback of their hand (represented by a cursor) was laterally shifted from trial to trial by an amount drawn from a skewed probability distribution. Skewed lateral shift probability distributions allowed us to separate the predictions of error-based and reinforcement-based loss functions on where to aim the hand. For example, a squared error loss function would predict that we should aim our hand to a location that corresponds to the mean of the skewed lateral shift probability distribution. Conversely, a reinforcement loss function would predict that we should aim our hand to a location that corresponds to the mode. Critically, skewed distributions separate several statistics, such as the mean and mode, that align with the optimal predictions of error-based and reinforcement based loss functions. Thus, by laterally shifting feedback using skewed noise and observing where participants reached, we were able to directly test how the sensorimotor system weights the relative influence of reinforcement feedback and error feedback when deciding where to aim the hand.

In Experiment 1 we tested how reinforcement feedback and error feedback influence where participants aimed their hand. We predicted that participants receiving only error feedback would minimize some form of error. We also predicted that participants receiving both error and reinforcement feedback would increasing rely on reinforcement feedback with a decrease in error feedback quality. Such a strategy predicts a different pattern of compensation for participants who received both forms of feedback when compared to those who only received error feedback. Surprisingly, we found that both error-only feedback and error plus reinforcement feedback resulted in participants minimizing approximately squared error. In Experiment 2 we used a modified task to verify that reinforcement feedback alone was capable of influencing where to aim the hand. Indeed, we found that participants who received only reinforcement feedback maximized the probability of hitting the target. However, we again found that participants minimized approximately squared error when both error and reinforcement feedback were present. Taken together, our results describe how the sensorimotor system uses error feedback and reinforcement feedback, in isolation and combination, when deciding where to aim the hand.

## Results

### Experiment 1

**Fig 1:**
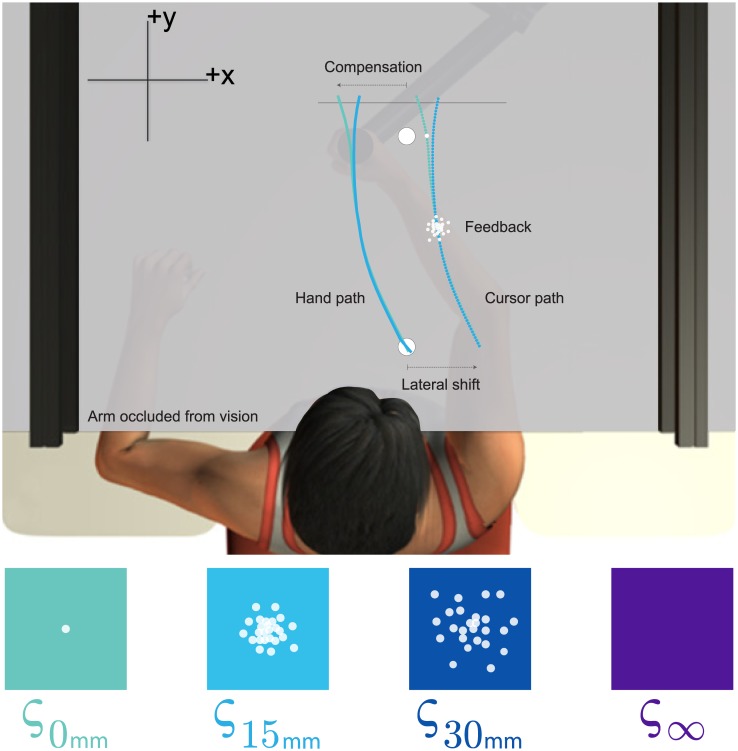
*Experiments 1 and 2*: Participants held the handle of a robotic arm with their right hand. A semi-silvered mirror reflected the image (visual targets, visual feedback) from an LCD screen (not shown) onto a horizontal plane aligned with the shoulder. Participants made forward reaches from a home position, attempted to move through a visual target and stopped once their hand passed over a horizontal line that disappeared when crossed. Error (visual) feedback and reinforcement (target expands, pleasant sound and monetary reward) feedback was laterally shifted relative to true hand position. The magnitude of any particular lateral shift was drawn from a skewed probability distribution. Participants had to compensate for the lateral shifts to hit the target. Compensation represents how laterally displaced their hand was relative to the displayed target. *Experiment 1*: Laterally shifted error feedback was flashed halfway through each reach as a single dot (*ς*_0*mm*_), a medium cloud of dots (*ς*_15*mm*_), a large cloud of dots (*ς*_30*mm*_), or withheld (*ς*_∞_). The cursor and hand (not visible) paths shown above illustrate compensation that depended on the amount of visual uncertainty (*ς*_0*mm*_ and *ς*_15*mm*_ conditions shown). In the single dot (*ς*_0*mm*_) condition, participants received additional feedback (error or error + reinforcement) at the target. *Experiment 2*: Participants were provided with error feedback and or reinforcement feedback only at the target.

**Fig 2:**
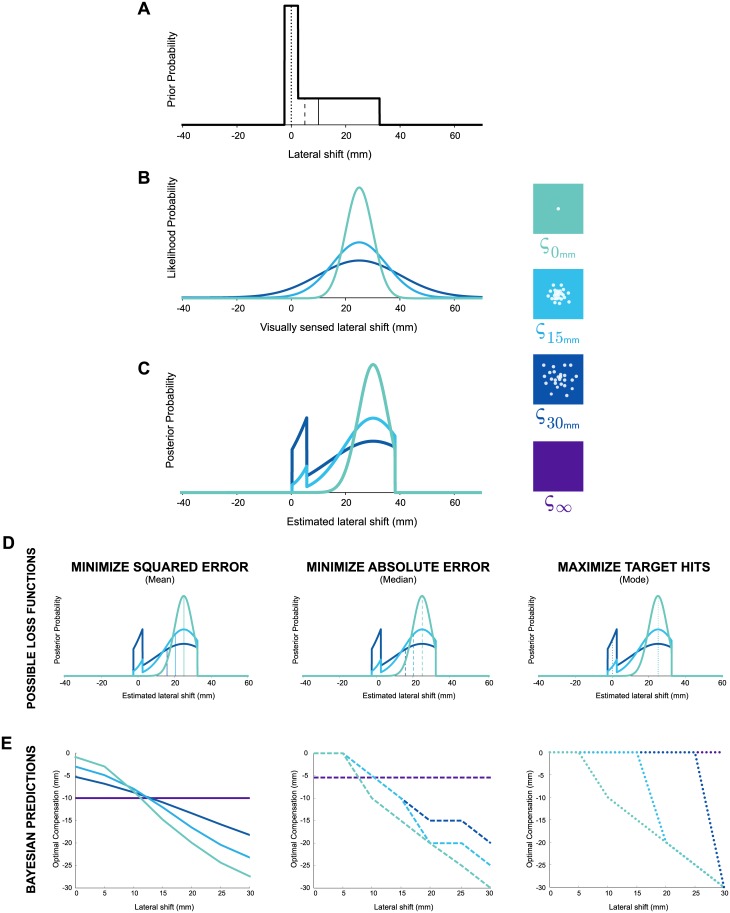
*Experiment 1*: By multiplying **A)** the probability of a lateral shift (prior probability) with **B)** the visually sensed lateral shift (likelihood probability) we obtain **C)** an estimate of the lateral shift (posterior probability) given current evidence and previous experience. The different colors in **B-E** represent the different amounts of visual uncertainty (*ς*_0*mm*_ = single dot; *ς*_15*mm*_ = medium cloud of dots; *ς*_30*mm*_ = large cloud of dots; or *ς*_∞_ = feedback withheld). For a lateral shift of 22.5*mm*, **D)** shows the optimal compensation for three different loss functions that either: i) minimize squared error, ii) minimize absolute error, or iii) maximize target hits, which is found by taking the mean (solid vertical line), median (dashed vertical line), or mode (dotted line) of a posterior, respectively. Differences between these strategies become more apparent with greater visual uncertainty. **E)** Optimal compensation for any lateral shift, given some level of visual uncertainty and a particular loss function. Note that the optimal compensation is equal to but in the opposite direction of the estimated lateral shift, representing a movement that counteracts a given lateral shift.

Participants performed 2000 reaching movements in a horizontal plane (Fig 1). They were instructed to “hit the target”. A cursor that represented the true hand position disappeared once the hand left the home position. On each trial, the unseen cursor was then laterally shifted by an amount drawn from a skewed probability distribution. Participants were randomly assigned to one of three groups (*n* = 10 per group). The *Error*_*SR*_ group and *Error*_*SL*_ group both received error feedback that was laterally shifted by a right-skewed (*RS*; Fig 2A) or left-skewed (*SL*) probability distribution, respectively. The third group, *Reinforcement* + *Error*_*SR*_, received error feedback and reinforcement feedback that were both laterally shifted by a *SR* probability distribution. Importantly, the skewed lateral shift probability distributions were designed to separate the mean and the mode, corresponding with the optimal solutions of error-based and reinforcement-based loss functions, respectively. This separation allowed us to investigate how the sensorimotor system weights the relative influence of reinforcement feedback and error feedback when deciding where to aim the hand. We hypothesized that with a decrease in error feedback quality the sensorimotor system would increase its reliance on reinforcement feedback.

To manipulate error feedback quality, halfway through each reach the laterally shifted cursor was either not presented (*ς*_∞_) or briefly presented (for 100*ms*) as a single dot (*ς*_0*mm*_), a medium cloud of dots (*ς*_15*mm*_) or a large cloud of dots (*ς*_30*mm*_), before disappearing once again (Figs 1 and 2). The medium and large clouds were composed of 25 dots, such that the dots were distributed according to a bivariate normal distribution with a standard deviation of 15*mm* and 30*mm*, respectively. Participants then attempted to hit the target by accounting for the laterally shifted error feedback (*ς*_0*mm*_, *ς*_15*mm*_, *ς*_30*mm*_, *ς*_∞_) they had experienced mid-reach. All participants received additional error feedback (also a single dot) at the end of the reach on trials in which single dot (*ς*_0*mm*_) error feedback was presented mid-reach [8].

Participants in the *Reinforcement* + *Error*_*SR*_ group were presented with reinforcement feedback only on trials in which the error feedback was presented as a single dot (*ς*_0*mm*_). The reinforcement feedback was binary (the target doubled in size, a pleasant sound was played over a loudspeaker, and participants received 2¢ CAD) and was presented when the laterally shifted cursor hit the target.

**Fig 3:**
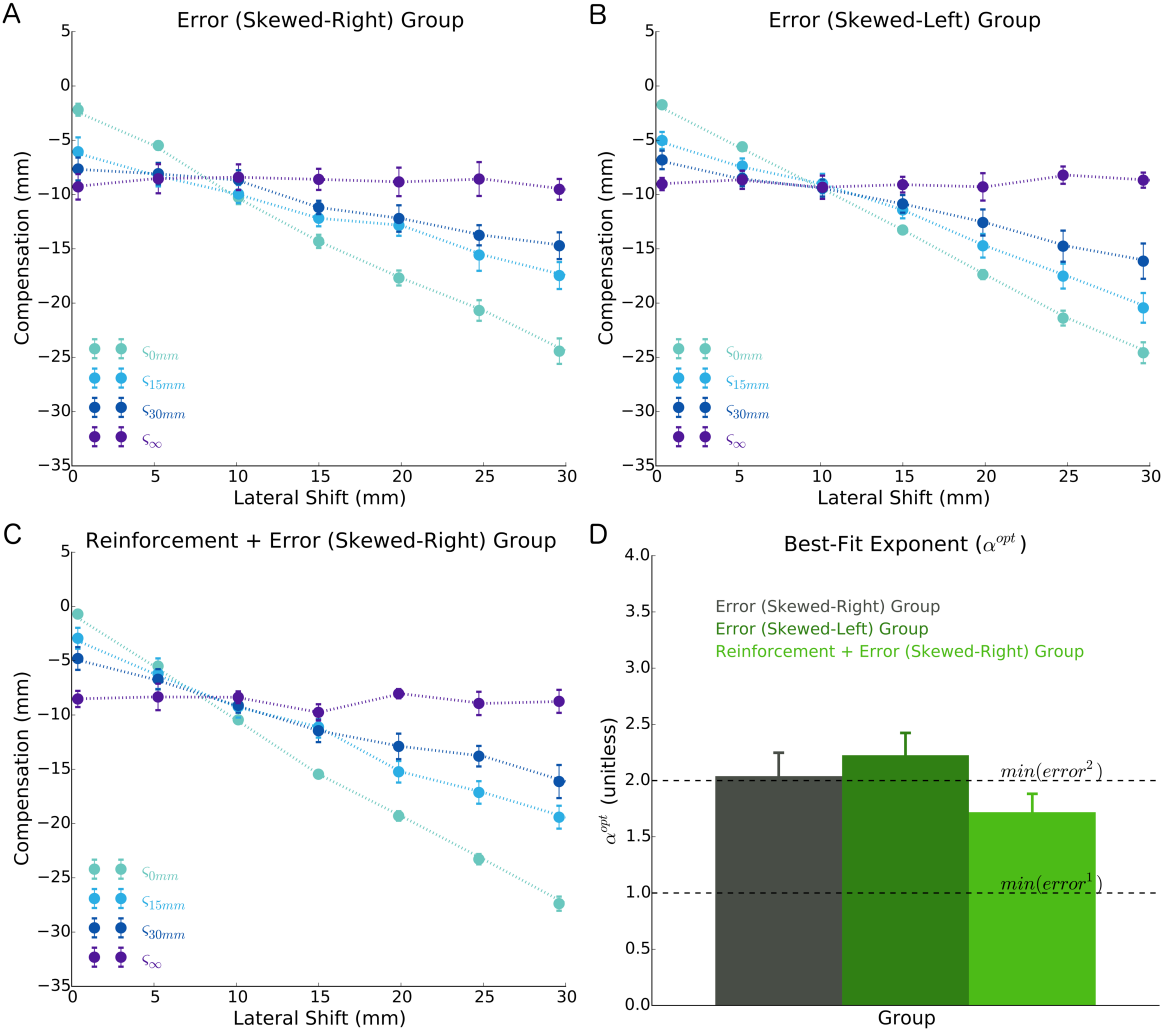
Average pattern of compensation (filled circles) in Experiment 1 to different magnitudes of lateral shift (*x*-axes) and visual uncertainty (separate lines) of participants receiving **A)** error feedback laterally shifted by skewed-right probability distribution, **B)** error feedback laterally shifted by a skewed-left probability distribution (note: these data are ‘flipped’ to visually align with the other groups), and **C)** both reinforcement and error feedback laterally shifted by a skewed-right probability distribution. A darker shade of blue signifies greater visual uncertainty. **D)** The best-fit power loss-function exponent (*α*^*opt*^) of a Bayesian model that, in addition to characterizing how error was minimized, was also a sensitive metric to whether participants were influenced by reinforcement feedback (see Methods). An exponent of 2.0 corresponds to minimizing squared error (upper dashed line), while an exponent of 1.0 corresponds to minimizing absolute error (lower dashed line). We found no significant differences in either the compensation (*p* = 0.956) or *α*^*opt*^ (*p* = 0.187) between groups. These findings suggest that all groups minimized approximately squared error, and that the sensorimotor system heavily weights error feedback over reinforcement feedback when both forms of feedback are available. Error bars represent ±1 standard error of the mean.

On each trial, we estimated how a participant compensated for the lateral shift by recording their hand location relative to the displayed target (see Fig 1). This was done for the four different levels of error feedback quality and lateral shift magnitudes. The average compensatory behavior of each group is shown in Fig 3. It can be seen that, with very little visual uncertainty about the magnitude of a lateral shift (*ς*_0*mm*_), all groups had a pattern of compensation that was well matched to the true magnitude of the shift. As error feedback quality decreased from little uncertainty (*ς*_0*mm*_) to some uncertainty (*ς*_15*mm*_ and *ς*_30*mm*_) to complete uncertainty (*ς*_∞_), participants’ pattern of compensation became increasingly less sensitive to the true magnitude of the lateral shift. This is consistent with Bayesian inference in that participants were increasing their reliance on their prior with a decrease in error feedback quality. Interestingly, the average compensation of each group, even for participants who received both reinforcement and error feedback, corresponded quite closely to the predictions made by a Bayesian model whose loss function minimized squared error (compare Figs 3A–3C to 2E).

To quantify the extent to which reinforcement feedback had an influence on behavior, we performed three separate analyses. First, we compared the average compensatory behavior between groups. Second, we used a Bayesian model to characterize how error feedback is used to guide behavior, and the extent to which reinforcement feedback influenced compensation. Finally, we used a simple linear model to characterize how the relationship between compensation and lateral shift is modulated by error signal quality, and the extent to which reinforcement feedback influenced compensation. All three analyses supported the idea that reinforcement feedback did not influence behavior when it was presented in combination with error feedback. Below, we describe each group’s compensatory behavior and the results of the Bayesian model. For brevity, detailed results of the linear model are presented in S2 Data.

#### Compensatory analysis

We compared average compensatory behavior between groups for the condition in which error feedback quality was lowest (*ς*_∞_). In this condition, there was the greatest separation between the predictions of a strategy that minimizes error and one that maximizes the probability of hitting the target (Fig 2E; **see dark blue lines**). Based on previous work [11, 14, 15], we expected participants that only received error feedback would minimize approximately squared error. Thus, we expected the *Error*_*SR*_ and *Error*_*SL*_ groups to show a pattern of compensation that corresponded closely to the mean of the skewed lateral shift probability distribution. Based on the hypothesis that there would be increased reliance on reinforcement feedback with a decrease in error feedback quality, we expected that when the error feedback quality was lowest, the *Reinforcement* + *Error*_*SR*_ participants would maximize target hits. Thus we expected these participants to approach a pattern of compensation that corresponded to the mode of the skewed lateral shift probability distribution. In summary, given the separation between the mean and mode of the skewed lateral shift probability distributions, we expected the *Reinforcement* + *Error*_*SR*_ group to have a significantly different pattern of compensation compared to the *Error*_*SR*_ and *Error*_*SL*_ groups.

Unexpectedly, we found no difference in the pattern of compensation between groups [*F*(2, 27) = 0.045, *p* = 0.956, ${\hat{\omega }}_{G}^{2}<0.001$]. The average compensation magnitude across groups was only ∼1*mm* away from the compensation location (−10*mm*) that minimized squared error (Fig 3A–3C, **dark blue lines**). This finding contradicts the hypothesis that the sensorimotor system would increase its reliance on reinforcement feedback with a decrease in error feedback quality. Rather, it suggests that the sensorimotor system heavily weights error feedback over reinforcement feedback when both are presented in combination.

#### Bayesian model

To model participant behavior and further assess the original hypothesis, we fit a Bayesian model to each participant’s data (Eqs 1–6). The model follows current putative theories of sensorimotor control and allows us to simultaneously consider all error feedback quality conditions (*ς*_0*mm*_, *ς*_15*mm*_, *ς*_30*mm*_, *ς*_∞_). Briefly, the model describes how participants combine previously acquired information about lateral shifts (prior) with sensed visual feedback (likelihood) to generate an estimate of the lateral shift (posterior). This lateral shift estimate is then used to determine the magnitude of compensation that counteracts a lateral shift. The model involves fitting four parameters. One parameter, *α*^*opt*^, describes how participants weight different magnitudes of error. The three remaining parameters, $\sigma_{sensed(1)}^{opt}$, $\sigma_{sensed(2)}^{opt}$, $\sigma_{sensed(3)}^{opt}$, characterize how participants integrate different levels of error feedback uncertainty (single dot, medium cloud and large cloud of dots) when deciding where to aim the hand.

Briefly, *α*^*opt*^ represents the exponent of a power loss function (Eq 3) that relates how errors of different magnitudes influence subsequent movements. Larger values of *α*^*opt*^ correspond to placing a greater emphasis on reducing large errors relative to small errors. Here, we used *α*^*opt*^ to characterize how error was minimized and also as a metric to test if reinforcement feedback influenced behavior. Given the distinct predictions for minimizing error and maximizing target hits (Fig 2E), *α*^*opt*^ becomes sensitive to whether reinforcement feedback had an influence on behaviour. Specifically, *α*^*opt*^ would have a lower value if reinforcement feedback had an influence on behavior. In this case, the pattern of compensation would correspond more closely to the mode of the skewed lateral shift probability distribution. This would signify that with a decrease in error feedback quality, participants’ compensation becomes increasingly driven by reinforcement feedback and less by error feedback.

We found that there were no significant differences in *α*^*opt*^ between groups [*F*(2, 27) = 1.786, *p* = 0.187, ${\hat{\omega }}_{G}^{2}=0.050$; Fig 3D]. Again, this contradicts the hypothesis that with decreases in error feedback quality the sensorimotor system increases its reliance on reinforcement feedback. The average *α*^*opt*^ across groups was 1.99 ± 0.11*SE*, which was significantly different from 1.0 (*p* < 0.001, two-tailed, $\hat{\theta }=100.0\%$) and not significantly different from 2.0 (*p* = 0.961, two-tailed, $\hat{\theta }=60.0\%$). An *α*^*opt*^ equal to 1.0 or 2.0 represents minimizing absolute error or minimizing squared error, respectively. Further, our *α*^*opt*^ estimate was not significantly different from the power-loss function with an exponent of 1.72 ± 0.008*SD* (*p* = 0.215, two-tailed; $\hat{\theta }=60.0\%$) reported by Körding and Wolpert (2004b). We refer the reader to S1 Data for further details about the Bayesian model fits.

#### Linear model

We used a simple linear model to characterize the relationship between lateral shift magnitude and compensation (hand position relative to the target). A separate fit was made for each level of error feedback quality (*ς*_0*mm*_, *ς*_15*mm*_, *ς*_30*mm*_, *ς*_∞_). For the intercepts and slopes of the linear model, we found that there were no statistically reliable differences between groups. As with the analyses described above, this is consistent with the idea that the sensorimotor system heavily weights error feedback over reinforcement feedback when both forms of feedback are available. S2 Data describes full details of this linear model.

Taken together, the three analyses reported above do not support the hypothesis that the sensorimotor system increases its reliance on reinforcement feedback with a decrease in error feedback quality. We found that participants minimized approximately squared error, even in the presence of reinforcement feedback that promoted maximizing the probability of hitting the target. This led to participants consistently missing the target when visual feedback was withheld. Moreover, it suggests that the sensorimotor system heavily weights error feedback over reinforcement feedback when guiding reaching movements.

### Experiment 2

In Experiment 1 we found that when both error feedback and reinforcement feedback were presented in combination, participant behavior seemed only driven by error feedback. There is a possibility that the reinforcement feedback we used was not capable of influencing behavior. To test this, in Experiment 2 some participants only received reinforcement feedback, without error feedback. It is important to note that these participants only received reinforcement feedback at the end of each movement. This represents a difference in experimental design from Experiment 1, whose participants (*Error*_*SR*_, *Error*_*SL*_, *Reinforcement* + *Error*_*SR*_) often received feedback twice in a single movement—mid-reach and as they passed by the target. To properly control for this, in Experiment 2 we tested two additional groups that received only error feedback, or error plus reinforcement feedback. Importantly, however, all participants in Experiment 2 only received feedback once per trial, at the end of movement. This ensured that the frequency and location of feedback received by the three groups was the same.

As a consequence of not providing error feedback mid-reach and only providing feedback at the target, compensation to the skewed lateral shift probability distribution in Experiment 2 reflects a trial-by-trial updating of where to aim the hand. This differs from Experiment 1 in which compensation reflects both online (via mid-reach feedback) and trial-by-trial (via target feedback) updating of where to aim the hand. In the context of a Bayesian framework, this would indicate in Experiment 2 that the prior representation of lateral shifts is updated after a trial is completed, instead of both during and after a trial is complete as in Experiment 1.

**Fig 4:**
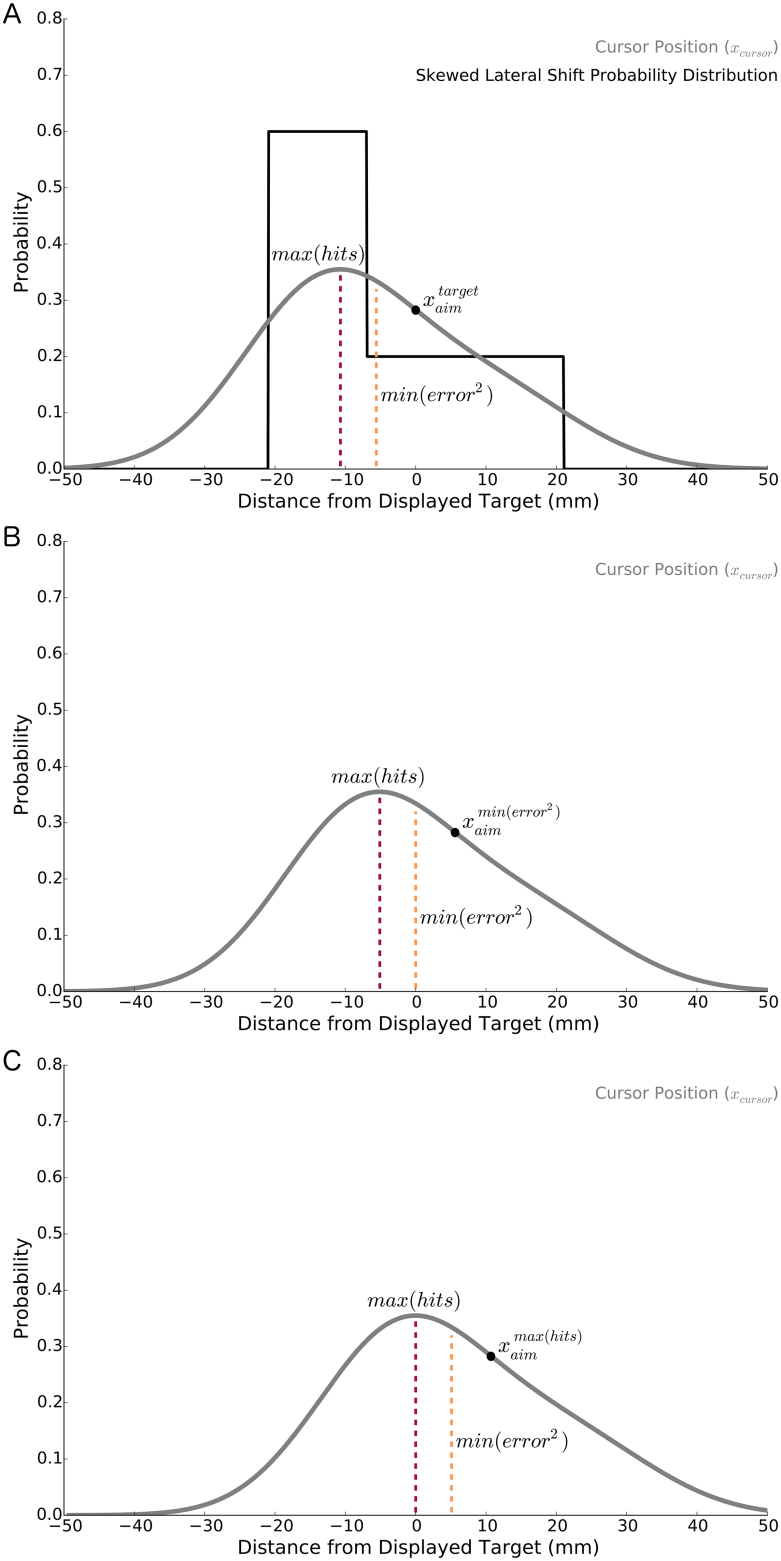
**A)** In Experiment 2 we used skewed probability distributions (black line, skewed-right distribution shown) to laterally shift the cursor from the true hand position. These lateral shifts were experienced by participants receiving error feedback, reinforcement feedback, or both forms of feedback. **A, B, C)** The *x*-axes represent the distance from the displayed target and the solid grey curve represents the probability of cursor position (*y*-axes) given intended aim of the hand, movement variability, and the skewed lateral shift probability distribution. The orange, dashed lines represent the mean of the cursor position probability distribution and corresponds to minimizing error [*min*(*error*^2^)]. The dark red, dashed lines represent the mode of the cursor position probability distribution and corresponds to maximizing target hits [*max*(*hits*)]. **A)** The probability of cursor position when the intended aim of the hand is aligned with the displayed target [$x_{aim}^{target}=0mm$]. **B)** The probability of cursor position when the aim of the hand minimizes square error [$x_{aim}^{min(error^{2})}=5.4mm$]. Notice that the mean (orange, dashed line) of the cursor position probability distribution is directly aligned with the displayed target. C) The probability of cursor position when the aim of the hand maximizes target hits [$x_{aim}^{max(hits)}=10.7mm$]. Here, the mode (dark red, dashed line) of the cursor position probability distribution is aligned with the displayed target.

One group of participants only received reinforcement feedback (*Reinforcement*), a second group received only error feedback (*Error*), and a third group received both error and reinforcement feedback (*Reinforcement* + *Error*). In total, there were ninety participants (30 per group). All participants performed 500 reaching movements in a horizontal plane. They were instructed to “hit the target”. On every trial, a cursor that represented the true hand position disappeared once the hand left the home position. The unseen cursor was laterally shifted by an amount drawn from a skewed-right (*SR*; *n* = 15 per group; Fig 4A) or skewed-left (*SL*, *n* = 15 per group) probability distribution.

Binary reinforcement feedback occurred when the laterally shifted cursor hit the target (the target doubled in size, a pleasant sound was played over a loudspeaker, and participants received 2 ¢ CAD). Error feedback was presented as a single dot at the end of the reach, at the location where the laterally shifted cursor passed by or through the target.

For each reach we recorded each participant’s pattern of compensation, that is, how laterally displaced his or her hand was relative to the displayed target (Fig 1). We calculated the compensation location that would maximize the probability of hitting the target ($x_{aim}^{max(hits)}$; Eqs 7, 8 and 12 and Fig 4C). This calculation incorporated a measure of movement variability at the target, which was larger in this experiment, relative to Experiment 1, given that there was no mid-reach error feedback (see Methods for further details). We also calculated the compensation location that would minimize squared error of cursor positions about the target ($x_{aim}^{min(error^{2})}$; Eqs 7–11 and Fig 4B).

**Fig 5:**
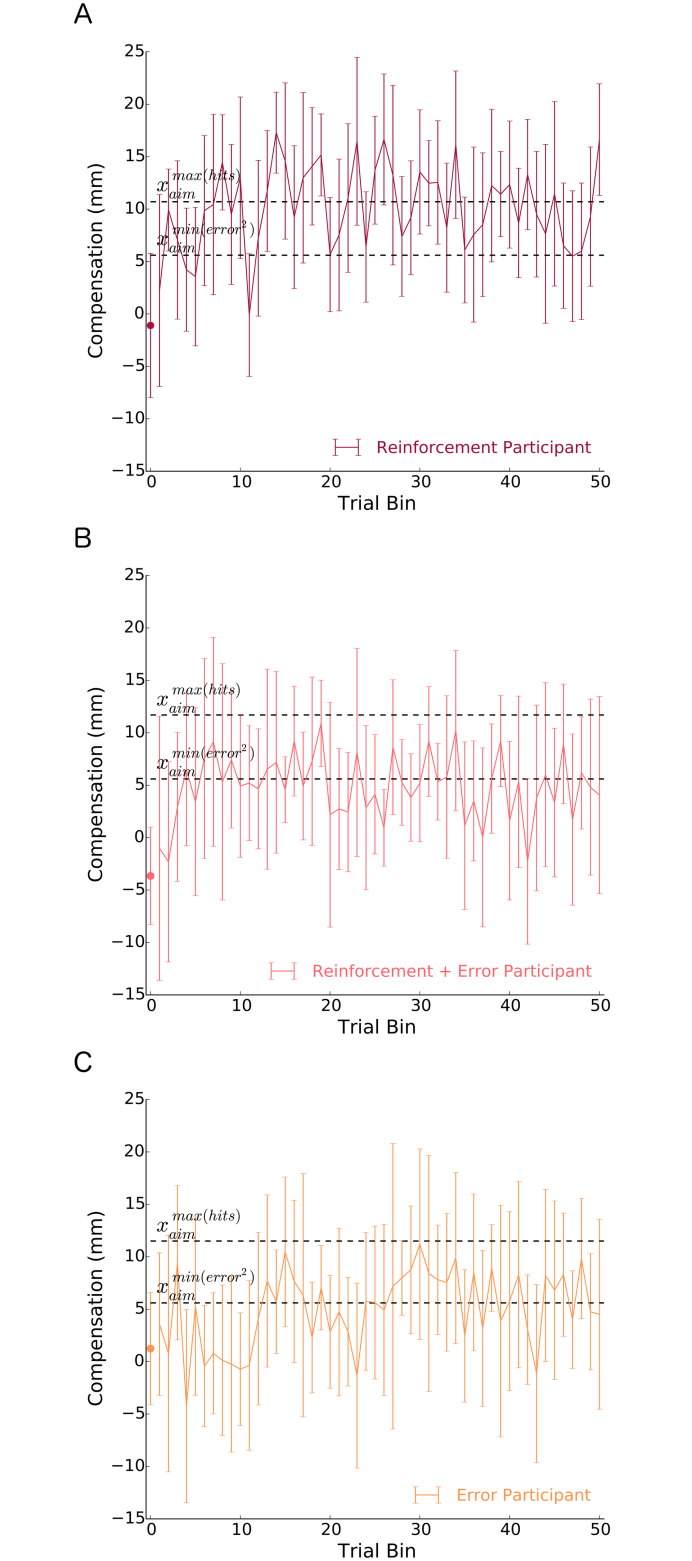
Pattern of compensation of a typical participant in the: A) reinforcement group, B) reinforcement + error group, and C) error group. All experimental trials are represented in the line graph (bins 1–50), where each point represents the average of 10 trials. The circles at bin 0 represent the average of the first three trials and show each participant’s behaviour immediately after perturbation onset, which was similar across groups (see Fig 6). For each group, the upper dashed line represents the optimal compensation (location to aim the hand) that maximizes the probability of hitting the target [$x_{aim}^{max(hits)}$]. The lower dashed line represents the optimal compensation (location to aim the hand) that minimizes squared error [$x_{aim}^{min(error^{2})}$]. It can be seen that the Reinforcement participant had a pattern of compensation that on average maximized target hits. Conversely, both the Error participant and the Reinforcement + Error participant learned a compensation that on average minimized approximately squared error. This behavior was consistent across participants. Error bars represent ±1 standard deviation.

Fig 5 shows the pattern of compensation of a participant from each group. In response to the skewed lateral shift probability distribution, it can be seen that the *Reinforcement* participant learned to compensate by an amount that was on average close to maximizing the probability of hitting the target. Conversely, both the *Error* and the *Reinforcement* + *Error* participants had an average compensation that corresponded to minimizing approximately squared error.

**Fig 6:**
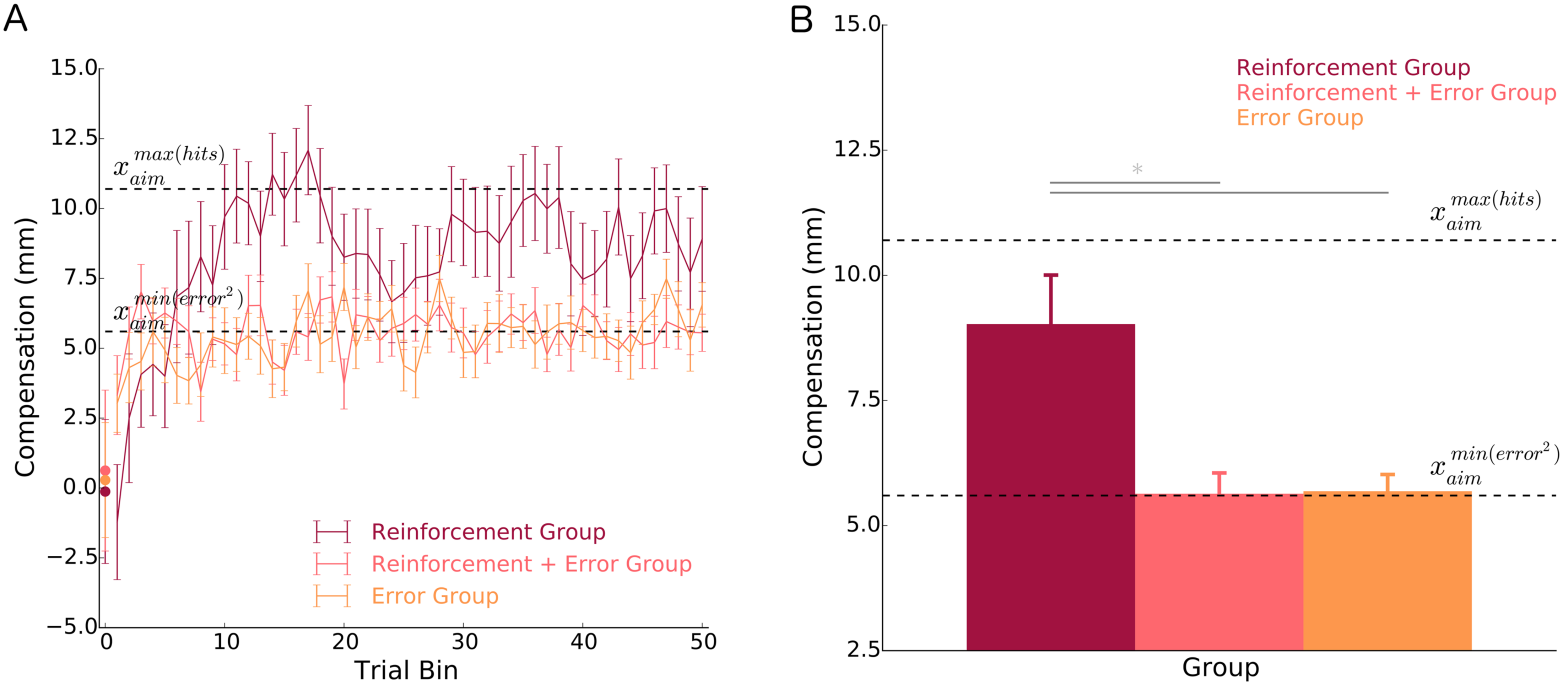
Average pattern of compensation of each group to the skewed lateral shift probability distribution for A) separate bins of trials and B) averaged across the last 400 trials. In **A)**, all experimental trials are represented in the line graph (bins 1–50), where each point represents the average of 10 trials. The circles at bin 0 represent the average of trials 1–3 and are displayed to show the similarity between groups immediately after perturbation onset. In both **A)** and **B)**, the upper dashed line represents the optimal compensation (location to aim the hand) that maximizes the probability of hitting the target $x_{aim}^{max(hits)}$, based on the movement variability of the Reinforcement group. The lower dashed line represents the optimal compensation (location to aim the hand) that minimizes squared error $x_{aim}^{min(error^{2})}$. These findings suggest that the sensorimotor system heavily weights error feedback over reinforcement feedback when both forms of feedback are available. Error bars represent ±1 standard error of the mean. * p < 0.05.

Fig 6A shows the average group compensatory behavior in response to the skewed lateral shift probability distribution. Compensation reached an asymptotic level after approximately 100 reaches (bin 10). Thus, for each participant, we averaged their last 400 trials to obtain a stable estimate of their behavior (Fig 6B). However, the results reported below were robust to whether we averaged the last 100, 200, 300 or 400 trials (Table 1).

**Table 1:** Group comparisons, p-values and effect sizes ($\hat{\theta }$), were robust to whether the last 100, 200, 300 and 400 trials were averaged together.

	Group Comparisons: p-value and effect size ($\hat{\theta }$)			
Binned Trials	Last 100 trials	Last 200 trials	Last 300 trials	Last 400 trials
*Reinforcement* vs. *Error*	*p* = 0.024, $\hat{\theta }=66.3$	*p* = 0.006, $\hat{\theta }=72.0$	*p* = 0.007, $\hat{\theta }=73.0$	*p* = 0.001, $\hat{\theta }=77.7$
*Reinforcement* vs. *Reinforcement* + *Error*	*p* = 0.023, $\hat{\theta }=66.3$	*p* = 0.005, $\hat{\theta }=73.1$	*p* = 0.010, $\hat{\theta }=73.0$	*p* = 0.004, $\hat{\theta }=76.7$
*Error* vs. *Reinforcement* + *Error*	*p* = 0.580, $\hat{\theta }=53.7$	*p* = 0.753, $\hat{\theta }=51.2$	*p* = 0.910, $\hat{\theta }=51.0$	*p* = 0.922, $\hat{\theta }=52.2$

To test whether the form of feedback and skew direction influenced behavior, we compared the average pattern of compensation between the three groups. We found that there was a significant main effect of group [*F*(2, 84) = 8.928, *p* < 0.001, ${\hat{\omega }}_{G}^{2}=0.150$]. There was no significant main effect of skew direction [*F*(1, 84) = 0.164, *p* = 0.687, ${\hat{\omega }}_{G}^{2}<0.001$] nor an interaction between group and skew direction [*F*(2, 84) = 0.498, *p* = 0.61, ${\hat{\omega }}_{G}^{2}<0.001$]. Thus, under the influence of the same skewed lateral shift probability distribution, we found that different forms of feedback resulted in significantly different compensatory behavior. Specifically, we found a statistically reliable difference between the *Reinforcement* and *Error* groups (*p* < 0.001, one-tailed; $\hat{\theta }=77.7\%$).

The *Reinforcement* group approached a compensatory position that would maximize their probability of hitting the target [$x_{aim}^{max(hits)}$] (*p* = 0.081, two-tailed; $\hat{\theta }=63.3\%$; *CI*[7.1, 10.8*mm*]). Further, the *Reinforcement* group’s pattern of compensation was significantly different from one that corresponded to the minimization of squared error [$x_{aim}^{min(error^{2})}$](*p* < 0.001, one-tailed; $\hat{\theta }=83.3\%$). These two findings suggest that the reinforcement feedback used in Experiments 1 and 2 is capable of influencing behavior in a way that aligns with a reinforcement-based loss function.

One prediction in the context of reinforcement-based learning is that an individual’s movement variability should influence their pattern of compensation to the lateral shifts. We tested this for the Reinforcement participants in Experiment 2. We characterized movement variability as the standard deviation of final hand position during the asymptotic phase of learning (the last 400 trials). While at the group level, participants appeared to compensate by an amount that approached an optimal strategy (see above), on an individual basis we did not find a statistically reliable relationship between movement variability and compensation (*R*^2^ = 0.003). This finding appears to differ from that reported by Trommershäuser et al. (2003a). However, as expanded on in the **Discussion**, there are many differences between their task and ours, and such findings are not uncommon [16, 25]. Nevertheless, the group level data suggests the existence of a reinforcement-based loss function that maximizes the probability of hitting the target.

As expected, the *Error* group minimized squared error (Fig 6B). Their pattern of compensation was aligned with one that, on average, minimized squared error (*p* = 0.795, two-tailed; $\hat{\theta }=50.0\%$). It was also significantly different from one that maximized their probability of hitting the target (*p* < 0.001, one-tailed; $\hat{\theta }=100.0\%$).

The results of Experiment 2 support the idea that the human sensorimotor system can update where to aim the hand during a reach by using only reinforcement-based feedback (to maximize the probability of hitting the target) or only error-based feedback (to minimize approximately squared error). As in Experiment 1, we again found that participants in the *Reinforcement* + *Error* group minimized squared error, and that reinforcement feedback did not influence behavior. Participants in the *Reinforcement* + *Error* group exhibited a pattern of compensation that was significantly different from the *Reinforcement* group (*p* = 0.004, two-tailed; $\hat{\theta }=76.7\%$), but was indistinguishable from participants in the *Error* group (*p* = 0.922, two-tailed; $\hat{\theta }=52.2\%$).

Taken together, the results from both Experiment 1 and 2 support the idea that the sensorimotor system heavily weights error feedback over reinforcement feedback when updating where to aim the hand during a reaching task.

## Discussion

A key aspect of this study was the use of skewed noise to separate the optimal aim locations predicted by reinforcement-based and error-based loss functions. This allowed us to probe how the sensorimotor system uses reinforcement feedback and error feedback, in isolation and combination, to update where to aim the hand during a reaching task. We found that participants minimized approximately squared error when they received only error feedback. Participants maximized the probability of hitting the target when they received only reinforcement feedback. When both forms of feedback were presented in combination, participants minimized approximately squared error at the expense of maximizing the probability of hitting the target. This finding suggests that the sensorimotor system heavily weights error feedback over reinforcement feedback when deciding to aim the hand.

In both Experiments, we found that the sensorimotor system minimized approximately squared error when using error feedback to guide reaching movements. This agrees well with previous work that examined how humans adapt to a small range of asymmetrical or multimodal noise during proprioceptive [11, 14] and visual [15] tasks. In Experiment 1 and 2, we also used small ranges of asymmetrical noise that respectively spanned 3*cm* and 2.8*cm* of the workspace. There is some evidence that as noise exceeds this range, the sensorimotor system is less sensitive to large errors [9, 26].

While in the present study our data points to an error-based loss function based on squared error, other studies have focused on other mathematical forms. Another commonly examined loss function is the inverted-Gaussian [9, 13, 16], which places greater emphasis on penalizing smaller errors and less emphasis on penalizing larger errors. Sensinger and colleagues (2015) used a biofeedback task that involved controlling a myoelectric signal corrupted with skewed noise similar to that used by Körding and Wolpert (2004b). They then examined several different loss functions and their corresponding best-fit parameters given the data. The parameters of a loss function define how errors of different sizes are weighted relative to one another. For the inverted-Gaussian loss function they found its best-fit parameter was much larger (9 times) than that found by Körding and Wolpert (2004b). They suggest that the inverted Gaussian loss function may not be generalizable across different motor tasks. They did, however, estimate a best-fit power loss function exponent of 1.69, a value that was quite close to the figure of 1.72 estimated by Körding and Wolpert (2004b). In the present study we estimated an average best-fit power loss function exponent closer to 2.0, which was not significantly different from 1.72. These differences may be due to the range of noise and possibility the shape of the skewed noise. Nevertheless, and similar to others [11, 14, 15], we were able to explain 80% to 89% of the variability in our data (see S1 Data) using a power loss function that minimized approximately squared error (i.e., *α*^*opt*^ ≈ 2.0).

In the current paper, we use a Bayesian framework to interpret and model how the sensorimotor system uses error feedback and reinforcement feedback when deciding where to aim the hand. This framework combines prior experience and current sensory information, such as sensory cues [27] and sensory uncertainty [8], in a statistically optimal fashion. By accounting for both prior and current information, the Bayesian framework has successfully explained a broad range of phenomena, such as reduced movement variability [8, 28], perceptual illusions [29] and online feedback control [30].

An alternative computational framework for error-based learning has been instrumental to our understanding of how the sensorimotor system learns to adapt on a trial-by-trial basis [31–35]. Of these models, the ones that account for sensorimotor noise [32, 33, 35] have been termed, ‘aim point correction’ models [36]. van Beers (2009) extended upon this framework with the ‘planned aim point correction’ model. This model separates central movement planning noise and peripheral movement execution noise. This model was able to explain reach adaptation patterns in a naturalistic task while demonstrating that the sensorimotor rate of learning was optimal given the properties of planning and execution noise. It has been successfully applied to explain differences in novice and expert dart throwers [37], and can account for both learning in task-relevant dimensions and exploratory (random walk) behaviour in task-irrelevant dimensions [38]. Aim point correction models, which can be derived from a Bayesian framework, are attractive because they are computationally tractable and learning is modeled using terms and constructs from sensorimotor control, such as planning noise, motor commands, efference copies, and execution noise [36]. In their current formulation, however, these models do not incorporate how the sensorimotor system responds to errors of differing magnitudes and different amounts of sensory uncertainty. We accounted for both of these factors with our Bayesian model, which was essential for testing our hypotheses. To further study how the sensorimotor system adapts on a trial-by-trial basis in experiments such as the ones used in this paper, a useful future direction would be incorporating the effects of sensory uncertainty and how errors of differing magnitudes are penalized.

While it is most common to study adaptation while providing only error feedback, researchers have also examined adaptation in the context of both reinforcement and error feedback [1, 3, 7, 12, 20–23, 39, 40]. Using a visuomotor rotation task, Izawa and Shadmehr (2011) examined how the sensorimotor system uses both a reinforcement and error feedback when deciding where to aim the hand during a reach. They manipulated the quality of error feedback presented on each trial in the following three ways: first, by displaying the cursor both at mid-reach and also at the target (mid-reach and target error-feedback condition); second, by displaying the cursor only at the target (target error-feedback condition); and thirdly, by withholding visual feedback of the cursor completely (no error-feedback condition). In each of these conditions, participants received reinforcement feedback for hitting the target (the target expanded and a pleasant sound was played over a loudspeaker). They modeled participants’ aiming behavior using a modified Kalman filter, which increasingly relied on reinforcement feedback as the quality of error feedback decreased. However, in the Izawa and Shadmehr (2011) experiment, the predicted aim location for minimizing error and maximizing the probability of hitting the target overlapped, making it difficult to determine the extent to which participants’ adaptation was driven by error feedback versus reinforcement feedback.

Izawa and Shadmehr (2011) found greater movement variability in trials that provided feedback only at the target when compared to trials that provided feedback both mid-reach and at the target. Their model attributed a greater proportion of adaptation due to reinforcement feedback on trials in which error feedback was provided at the target, compared to trials in which error feedback was provided both mid-reach and at the target. While this is certainly a possibility, an alternative explanation for these behavioral differences is that the participants receiving feedback both mid-reach and at the target, unlike those receiving feedback only at the target, were able to compensate for accumulated sensorimotor noise error they sensed mid-reach. That is, it is difficult to determine whether behavioral differences between conditions were a result of reinforcement feedback, differences in the amount of (or location of) feedback, or both.

In the present study we designed experiments aimed at resolving both of these potential issues. First, we were able to separate the optimal aim location of error-based and reinforcement-based loss functions. Second, for each group in Experiment 2 we equated the amount of and location of feedback by providing it only at the target. We found no differences in compensation between participants who received error feedback and participants who received both error and reinforcement feedback. This finding aligns with the work of Vaswani et al. (2015), who similarly found that reinforcement feedback did not influence behaviour when it was combined with error feedback. This suggests that the sensorimotor system heavily weights error feedback, when available, over competing reinforcement feedback.

Trommershäuser and colleagues (2003a) provided evidence that humans can use reinforcement feedback to adjust where they aim their hand during a reaching task. In their study, participants reached to a screen displaying a rewarding target area (positive reinforcement: monetary gain) and an overlapping penalty area (negative reinforcement: monetary loss). Thus, participants received both reinforcement feedback for hitting the target, and error feedback that indicated where they touched the screen relative to the target. The reward and penalty for hitting these respective areas were held constant for a given block of trials. In their task, internal sensorimotor noise created a smooth and differentiable reinforcement landscape within and surrounding the reward and penalty areas. They found participants accounted for their internal sensorimotor noise and aimed towards the peak of this reinforcement landscape to maximize the probability of hitting the target. In our task, we found that participants receiving only reinforcement feedback maximized the probability of hitting the target, but participants who received both reinforcement and error feedback minimized squared error.

Here, we suggest two potential reasons why participants receiving both reinforcement and error feedback continued to minimize error in our task, and appeared not to be driven by reinforcement feedback. First, it may be related to how the reinforcement region was spatially defined [10]. Second, the finding may only be present during implicit learning [41]. In the Trommershäuser et al. (2003a) experiments, the reinforcement regions were static and clearly defined in the workplace by the bounds of the penalty and reward areas. Thus, participants may have used error feedback to guide their aim towards the location they explicitly learned would maximize the probability of hitting the target. In our task, a stationary target was displayed on the screen. However, due to the lateral shifts, this target did not always represent the true reinforcement region. Moreover, similar to other experiments that used a small range of external noise and in which participants were unaware of their average change in reaching aim direction [8], our task was most likely implicitly learned [41]. Thus, unlike Trommershäuser et al. (2003a), our participants may have had to implicitly build a representation of the probabilistically varying reinforcement regions.

In the absence of any error feedback, we found that participants receiving only reinforcement feedback compensated by an amount that approached the optimal compensation that maximized the probability of hitting the target. In accordance with a loss function that maximizes the probability of hitting the target, this suggests that the sensorimotor system was able to learn and compensate for the probabilistically provided reinforcement feedback. Despite an average compensation across participants that did not differ from the optimal solution, we did not find a correlation between final compensation and movement variability on an individual basis. This differs from Trommershäuser et al. (2003a), but as mentioned above there are many differences in experiment design between their task and ours. However, optimal performance across participants with suboptimal performance on an individual basis has been previously reported when using complex distributions (i.e., skewed or bimodal distributions) to perturb feedback [16, 25]. From a probabilistic viewpoint, suboptimal performance on an individual basis may partially be explained by individual differences in exploratory movement variability [42], as well as inaccurate [10], approximate [15] or stochastic [16] representations of the prior, likelihood and posterior.

To produce accurate goal-directed movements, our nervous system must account for uncertainty and nonlinearities present in our environment [8, 9, 11], in the biomechanics of our body [43, 44], and in our nervous system [6, 45–47]. By using skewed probability distributions, we were able to separate the optimal aim locations predicted by reinforcement-based and error-based loss functions. The results of our experiments demonstrate that by changing the form of feedback provided to participants, reinforcement-based and error-based learning are dissociable and can occur independently. Further, we also found that when both a reinforcement and error feedback are available, the sensorimotor system relies heavily, perhaps exclusively, on error feedback when deciding where to aim the hand during targeted reaching. Our work consolidates and builds upon previous research to provide insight into how the sensorimotor system performs model-based and model-free learning.

## Methods

### Participants

30 individuals (20.2 ± 2.7 years) participated in Experiment 1 and 90 individuals (20.6 ± 2.4 years) participated in Experiment 2. All participants were healthy, right-handed and provided informed consent to procedures approved by Western University’s Ethics Board.

### Apparatus

In both experiments, participants performed right-handed reaching movements in a horizontal plane while grasping the handle of a robotic arm (InMotion2, Interactive Motion Technologies, Massachusetts, United States; Fig 1). A semi-silvered mirror occluded vision of the arm and projected images from an LCD screen onto a horizontal plane aligned with the shoulder. These images included visual targets and, in certain conditions, real-time visual feedback of either the true or laterally shifted hand position. The workspace was calibrated such that the centroid of unperturbed visual feedback matched the center of the robotic handle. An air-sled supported each participant’s right arm and provided minimal friction with the desk surface. An algorithm controlled the robot’s torque motors and compensated for the dynamical properties of the robotic arm. Hand position was recorded at 600*Hz* and stored for offline analysis.

### Protocol

#### Experiment 1

On each trial, participants either received error feedback, or both error and reinforcement feedback. Participants were randomly assigned to one of the following three groups: 1) error, skewed-right group (*Error*_*SR*_), 2) error, skewed-left group (*Error*_*SL*_), and 3) reinforcement plus error, skewed-right group (*Reinforcement* + *Error*_*SR*_). Superscripts indicate the skewed lateral shift probability distribution from which laterally shifts were drawn on a trial-to-trial basis. Skew direction was skewed-right (*SR*) or skewed-left (*SL*).

Each participant performed 2000 reaching movements. For each trial, participants started from a home position, reached through a target, and stopped once the robot handle passed over a horizontal line that disappeared when crossed (Fig 1). The diameter of both the home position and the target was 0.5*cm*. We instructed participants to “hit the target”. To ensure that participants able to adequately adjust for error feedback received mid-reach, we instructed participants to move at a comfortable pace that was greater than 0.5*sec* per reach. If participants moved too quickly, the home position, target and horizontal line all turned from blue to red and they were instructed to slow down their movement.

When participants left the home position the cursor representing their true hand position disappeared. Once the cursor disappeared, its location was laterally shifted along the *x*-axis (Fig 1) using a magnitude drawn from a skewed probability distribution (*SR* or *SL*). After participants had moved forward 10*cm*, halfway between the home position and target, the laterally shifted cursor briefly reappeared for 100*ms* and then disappeared again (as in [8]). To experimentally manipulate visual uncertainty (error feedback quality), this mid-reach error feedback was presented on any given trial either as a single dot (*ς*_0*mm*_), a medium cloud of 25 dot (*ς*_15*mm*_), a large (*ς*_30*mm*_) cloud of 25 dots, or withheld (*ς*_∞_). The bivariate normally distributed x- and y-coordinates of the 25 dots used for the medium and large clouds had a standard deviation of 15 and 30*mm*, respectively, and were generated using the Box-Muller transformation [48]. The presentation of these 4 types of error feedback was randomized across trials. The relative presentation frequency of the four types of error feedback, *ς*_0*mm*_, *ς*_15*mm*_, *ς*_30*mm*_, *ς*_∞_, followed a 3: 1: 1: 1 ratio, respectively. For each trial, the centroid of the laterally shifted cursor represented true hand position plus a lateral shift drawn from one of the skewed probability distributions described above.

In Experiment 1, the *SR* (mean = 10.0*mm*, median = 5*mm*, mode = 0.0*mm*, range = 0.0 to 30*mm*; Fig 2A) and *SL* (mean = 10*mm*, median = 15*mm*, mode = 20.0*mm*, range = −10 to 20*mm*) probability distributions were designed so that the mean was the same (10*mm*) and the modes were on the opposite sides of the mean (0.0 and 20*mm*, respectively). Further, we designed these probability distributions such that the modal lateral shift occurred with a much higher relative frequency (42.8%) than the other six lateral shifts (9.5%). This difference in frequency increased the possibility of the sensorimotor system being able to distinguish the mode from the other lateral shifts. Lateral shift magnitude was randomly sampled from a skewed probability distribution with replacement [8]. Opposite skew directions (left versus right) assured that biomechanical (e.g., joint comfort, interaction torques, control effort, etc.) or other potential factors did not influence the results by causing a systemic left or right bias in reaches. That is, we controlled for spurious biases to be assured that participants were on average compensating for a lateral shift estimate that lay somewhere close to or within the bounds of the mean and mode of the skewed lateral shift probability distributions, regardless of skew direction.

For the full vision condition (*ς*_0*mm*_), participants from all groups received error feedback (single dot) as their hand passed by the target. This error feedback was provided along the *x*-axis that passed through the target and was presented using the same lateral shift as the error feedback provided mid-reach. By using only *x*-position feedback at the target, we reduced the task goal and subsequent analysis to one dimension. In addition to the target error feedback, the *Reinforcement* + *Error*_*SR*_ group also received binary reinforcement feedback when the laterally shifted cursor hit the target [1]. This feedback included visual (target briefly doubled in size), auditory (pleasant sound) and monetary (2 ¢ CAD) components.

By providing error and reinforcement feedback at the target only in the full vision condition, we were able to assess whether participants were: a) learning a mapping between visual feedback to an estimate of a lateral shift or b) building a representation of the skewed lateral shift noise distribution (i.e., a prior) [8]. If participants were simply using a mapping between visual feedback and lateral shifts, they could only do so for the full vision condition (*ς*_0*mm*_) and apply this same mapping to the medium (*ς*_15*mm*_) and large (*ς*_30*mm*_) cloud conditions. In Experiment 1, such a mapping would cause participants to compensate similarly for all three conditions (*ς*_0*mm*_, *ς*_15*mm*_, *ς*_30*mm*_), despite varying levels of error feedback uncertainty. In other words, they *would not* combine previous experience with current evidence, making them unable to produce a statistically optimal estimate of any given lateral shift. Alternatively, participants could build up a representation of the underlying probability distribution used to specify lateral shifts, and compensate in a way that aligns with a Bayesian inference. That is, they would combine previous experience (prior) with current information (likelihood) to produce a statistically optimal estimate (posterior) of any given lateral shift. In Experiment 1, this would be behaviorally expressed as a different pattern of compensation for each of the three conditions (*ς*_0*mm*_, *ς*_15*mm*_, *ς*_30*mm*_), which are characterized by different levels of error feedback uncertainty.

Once a participant’s hand stopped past the horizontal line, this line disappeared. The robot then moved their hand from its stopped position back to the home position in one second, using a minimum jerk (3^rd^ derivative of position) trajectory [49]. We scheduled 1*min* breaks every 250 trials to reduce fatigue.

#### Experiment 2

Here we used a modified version of the task used in Experiment 1 that allowed us to 1) verify that the sensorimotor system can update where to aim the hand when using only probabilistically provided reinforcement feedback, 2) to control for the frequency and location of feedback across groups, and 3) replicate the finding that the sensorimotor system heavily weights error feedback over reinforcement feedback.

90 participants either received reinforcement feedback, error feedback, or both. They were randomly assigned to and equally divided among the following three groups: 1) reinforcement group (*Reinforcement*), 2) error group (*Error*), and 3) reinforcement plus error group (*Reinforcement* + *Error*). For each group, an equal number of participants received error and or reinforcement feedback that were laterally shifted with a skewed-right (*SR*) or skewed-left (*SL*) probability distribution.

Each participant performed 500 forward reaches to a target. Participants started from a home position, attempted to reach through a target 20*cm* away, continued 2 more *cm* and stopped once they passed over a horizontal line that disappeared once crossed. We instructed all participants, “to hit the target, to move at a comfortable pace, and that it is a very difficult task and impossible to hit the target every time”.

Cursor position, whether visible (error feedback) or not visible (pure reinforcement feedback), was again laterally shifted from the true hand position from trial to trial by an amount drawn from a skewed probability distribution (i.e., cursor position = true hand position + random lateral shift). In Experiment 2, the two skewed lateral shift probability distributions, *SR* (Fig 4; mean = −5.6*mm*, median = −14.0*mm*, mode = −14.0*mm*, range = −14.0 to 14.0*mm*) and SL (mean = 5.6*mm*, median = 14.0*mm*, mode = 14.0*mm*, range = −14.0 to 14.0*mm*), had the same shape but opposite skew. These distributions each contained 500 lateral shifts that were randomly sampled until depletion. The modal lateral shift was presented with a frequency of 60% (300 trials) and the remaining two lateral shifts were presented on 20% of trials (100 trials each). Both distributions were characterized by three lateral shift values of −14.0*mm*, 0.0*mm*, and 14.0*mm*.

The spacing between the three lateral shifts was determined by considering the average movement variability of ten pilot participants who made reaches void of any feedback. By matching the spacing between lateral shifts to movement variability, we were able to ensure that participants were naturally exposed to the different regions of the workspace that provided positive reinforcement. This design was critical for participants in the *Reinforcement* group, since they did not have the aid of error feedback to help guide their reaches. The target diameter (14*mm*) matched the spacing between adjacent lateral shifts.

In Experiment 2, feedback was only provided along a lateral axis that passed through the target (Fig 1). For the *Error* and *Reinforcement* + *Error* groups, error feedback was a single dot that appeared along the *x*-axis where the laterally shifted cursor passed by or through the target. The *Reinforcement* and *Reinforcement* + *Error* groups received reinforcement feedback when the displaced cursor hit the target (within 14*mm*). This reinforcement feedback included visual (target briefly doubled in size), auditory (pleasant sound) and monetary (2 ¢ CAD) components [1, 17, 50]. At the end of each movement, the robot moved their hand back to the home position using a minimum jerk trajectory [49].

### Data analysis

We performed all offline analyses with custom Python (2.7.9) scripts.

#### Experiment 1

For each of the 2000 trials, we recorded how participants compensated, that is, their *x*-coordinate position where the hand passed through or by the target (Fig 1), given the error feedback quality and the magnitude of the lateral shifted feedback. We analyzed the last 1000 reaches to ensure that participants had sufficient training to learn the underlying lateral shift distribution. For each participant, we found their average compensation for all 28 combinations of the seven different lateral shifts (*i*) and four different levels of visual uncertainty (*j*).

To make visual and statistical comparisons regardless of skew, the recorded compensation data for participants in the *Error*_*SL*_ were ‘flipped’ about the aligned position (here, the mean) of the two skewed lateral shift probability distributions [16]. In Experiment 1, if the *SL* distribution were rotated about this position it would be identical to the *SR* distrubtion.

We used a Bayesian model to calculate participant’s estimate of their lateral shift halfway through a reach (Eq 1). By multiplying the probability of a true lateral shift [prior probability = *p*(*x*_*shift*(*i*)_)] with the probability of a participant’s visually sensed lateral shift [likelihood probability = *p*(*x*_*sensed*(*i*)_|*x*_*shift*(*i*)_, *σ*_*sensed*(*j*)_)] that is based on their mid-reach error feedback, we obtained the probability of their estimated lateral shift along the *x*-axis [posterior probability = *p*(*x*_*shift*(*i*)_|*x*_*sensed*(*i*)_, *σ*_*sensed*(*j*)_)]. This is summarized by

$$p\left(x_{shift(i)}|x_{sensed(i)},\sigma_{sensed(j)}\right)\propto p\left(x_{sensed(i)}|x_{shift(i)},\sigma_{sensed(j)}\right)\cdot p\left(x_{shift(i)}\right),$$ (1)

where ∝ represents proportionality between both sides of the equation, ⋅ indicates a point-wise multiplication between probability distributions, *i* represents some lateral shift, and *j* represents some amount of visual uncertainty. *x*_*sensed*(*i*)_ and *σ*_*sensed*(*j*)_ are a participant’s estimate of the visual feedback centroid and the uncertainty associated with some lateral shift, respectively. Like Körding and Wolpert (2004a), we assumed that *x*_*sensed*(*i*)_ matched the centroid of the lateral shift and that *p*(*x*_*sensed*(*i*)_|*x*_*shift*(*i*)_, *σ*_*sensed*(*j*)_) was normally distributed:

$$p\left(x_{sensed(i)}|x_{shift(i)},\sigma_{sensed(j)}\right)=\frac{1}{\sigma_{sensed(j)}\sqrt{2\pi }}\;e^{-\frac{{\left(x_{shift(i)}-x_{sensed(i)}\right)}^{2}}{2\sigma_{sensed(j)}^{2}}}.$$ (2)

Eq 1 was calculated numerically.

The posterior represents the probability of all possible lateral shifts halfway through the reach. The nervous system must select one action while considering many possible lateral shifts, weighted by their probability. The optimal action selection depends on the posterior and some strategy reflecting the goal(s) of the nervous system. For example, participants could compensate by an amount that aligns with the mode of the posterior (maximum a posteriori) if the sensorimotor system was maximizing the probability of hitting the target. Alternatively, participants could compensate by an amount that aligns with the median or mean of the posterior to minimize absolute or squared error, respectively. Thus, given previous experience, current information and a given strategy, we can find the “point estimate” from the posterior that aligns with the statistically optimal compensation ($comp_{i,j}^{opt}$) (Eq 5).

We used a Bayesian model (Eqs 1 and 2) to predict what participants’ compensation would be if they attempted to maximize success (number of target hits), minimize absolute error or minimize squared error by finding the mode, median and mean of the posteriors, respectively. The results of this simulation are shown in Fig 2.

Upon visually examining the data, we found that all groups appeared to be minimizing some form of error (e.g., compare Figs 2E to 3A–3C). To test for differences between groups, we used the following power loss function to model each participant’s behavior [9]:

$$L\left(x_{shift(i)},x_{comp},\alpha \right)={\left|x_{shift(i)}-x_{comp}\right|}^{\alpha },$$ (3)

where || takes the absolute value of the operations contained within. The exponent *α* is free to vary and defines how a participant minimizes error. The cost of compensating for any particular amount (*x*_*comp*_) given some lateral shift can be calculated with the expected loss function,

$$EL\left(x_{comp},\alpha ,x_{sensed(i)},\sigma_{sensed(j)}\right)=\int_{-\infty }^{\infty }L\left(x_{shift(i)},x_{comp},\alpha \right)p\left(x_{shift(i)}|x_{sensed(i)},\sigma_{sensed(j)}\right)dx_{shift(i)}.$$ (4)

The statistically optimal compensation ($comp_{i,j}^{opt}$) is found by minimizing the expected loss function,

$$comp_{i,j}^{opt}=-\underset{x_{comp}}{arg min}[EL(x_{comp},\alpha ,x_{sensed(i)},\sigma_{sensed(j)})].$$ (5)

The negative sign indicates that the optimal compensation is equal to but in the opposite direction of the estimated amount to compensate for (i.e., the estimated lateral shift). In the special cases where *α* equals 1 or 2, $comp_{i,j}^{opt}$ corresponds to a lateral shift estimate that is precisely aligned with the median or mean of the posterior distribution, respectively.

We fit the Bayesian model to each participant’s behavioral data. Model inputs for each participant were their compensation at the target ($comp_{i,j}^{opt}$), the centroid of visual feedback (*x*_*sensed*(*i*)_), and a weighting factor (*w*_*i*,*j*_). The weighting term allowed us to equally weight the four different conditions during the fitting procedure, while placing proportional emphasis on behavioral data according to the associated frequency of a particular lateral shift. The four model parameters provided an estimate of each participant’s error minimization strategy (*α*^*opt*^) and their uncertainty ($\sigma_{sensed(1)}^{opt}$, $\sigma_{sensed(2)}^{opt}$, $\sigma_{sensed(3)}^{opt}$) for the single dot, medium cloud and large cloud of dots, respectively. It was assumed that participants’ uncertainty with visual feedback withheld approached infinity ($\sigma_{sensed(4)}^{opt}\to \infty$), which would be physically represented as no changes in compensation with different lateral shifts (see the dark blue, horizontal lines in Fig 2E). We estimated the four model parameters using a Nelder-Mead optimization routine contained in the SciPy module (optimize, minimization) in Python [51]. We used least absolute error to find the four best-fit model parameters, which is a more robust fitting procedure than least squared error [52]. This procedure is summarized by

$$\alpha^{opt},\sigma_{sensed(1)}^{opt},\sigma_{sensed(2)}^{opt},\sigma_{sensed(3)}^{opt}=\underset{\alpha ,\sigma_{sensed(j)}}{arg min}[\sum_{i=1}^{7}\sum_{j=1}^{4}w_{i,j}\cdot \left|comp_{i,j}^{data}-comp_{i,j}^{opt}\right|].$$ (6)

When visual error feedback was withheld (*ς*_∞_), we would expect the *Reinforcement* + *Error*_*SR*_ group to have an average compensation that corresponded closely to the mode of the skewed lateral shift probability distribution, and to be significantly different from the *Error*_*SR*_ and *Error*_*SL*_ groups. We used *α*^*opt*^ to characterize how error was minimized and also as a metric to test if reinforcement feedback influenced behavior. Given the distinct optimal compensations that correspond to minimizing error and maximizing the probability of success (Fig 2E), *α*^*opt*^ is a sensitive metric to whether the reinforcement feedback had an influence on behaviour. Specifically, if the reinforcement feedback had an influence on behavior, we would find a lower *α*^*opt*^ for the *Reinforcement* + *Error*_*SR*_ group, relative to the *Error*_*SR*_ and *Error*_*SL*_ groups, as their average compensation would correspond more closely to the mode of the skewed lateral shift probability distribution (i.e., the mode of the posterior probability distribution). This would signify that, with a decrease in error feedback quality, participants’ compensation was becoming increasingly driven by reinforcement feedback and less by error feedback.

#### Experiment 2

For each reach we recorded how a participant compensated, that is, how laterally displaced their hand was when they passed by or through the displayed target, in response to the skewed lateral shift probability distribution (Fig 1). We also characterized each participant’s hand movement variability as the standard deviation of their final hand position during the asymptotic phase of learning (the last 400 trials). As in Experiment 1, we ‘flipped’ the recorded compensation data about the aligned position of the two skewed lateral shift probability distributions (here, 0*mm*) for any participant for whom feedback was shifted by the *SL* distribution.

For each group, we estimated the optimal location to aim the hand that minimized squared error and the optimal location to aim the hand that maximized target hits (Fig 6). To do this, we first modeled the probability of cursor position along the *x*-axis [*p*(*x*_*cursor*_)] by considering uncertainty in hand position [*p*(*x*_*hand*_)] and the skewed lateral shift probability distribution [*p*(*x*_*shift*(*i*)_) = *SR* or *SL*]. As a reminder, cursor position was not visible for the *Reinforcement* group and visible for both the *Error* and *Reinforcement* + *Error* Groups. Since *p*(*x*_*hand*_) and *p*(*x*_*shift*(*i*)_) are independent random processes, we numerically convolved (*) these two probability distributions to find their additive effect on cursor position [*p*(*x*_*cursor*_)] as follows:

$$p\left(x_{cursor}|x_{aim},\sigma_{mv}\right)=p\left(x_{hand}|x_{aim},\sigma_{mv}\right)*p\left(x_{shift(i)}\right).$$ (7)

Hand position [*p*(*x*_*hand*_)] was modeled with a Normal probability distribution [7, 21, 53],

$$p\left(x_{hand}|x_{aim},\sigma_{mv}\right)=\frac{1}{\sigma_{mv}\sqrt{2\pi }}\;e^{-\frac{{\left(x_{hand}-x_{aim}\right)}^{2}}{2\sigma_{mv}^{2}}},$$ (8)

where *x*_*aim*_ represents an unbiased hand aim location and *σ*_*mv*_(*mm*) is the average hand movement variability of participants in a group (*Reinforcement* = 10.6*mm*, *Error* = 9.2*mm*, *Reinforcement* + *Error* = 8.8*mm*). Movement variability for all three groups was slightly greater than that of a pilot group (7.0*mm*) who reached to the displayed target with no feedback whatsoever. Relative to the pilot group, the increase in variability for participants receiving error feedback is likely related to them compensating to some extent for error feedback on a trial by trial basis. For participants receiving only reinforcement feedback, their increase in movement variability may be attributed to exploration [54]. Additional exploration has been suggested as a way to increase the rate of learning [42, 55]. We refer the reader to Fig 4A, which shows the skewed lateral shift probability distribution and the probability of cursor position when the hand is aimed at the displayed target.

With our model of cursor position, using Eqs 9–11 we then calculated the optimal location to aim the hand that minimizes the squared error of possible cursor positions relative to the displayed target (*x*_*target*_; set to 0 for all simulations). First, we define the squared error loss function,

$$L\left(x_{cursor},x_{target}\right)={\left|x_{cursor}-x_{target}\right|}^{2}.$$ (9)

Then, the squared error cost of any cursor position relative to the displayed target can be calculated by the following expected loss function:

$$EL\left(x_{aim},\sigma_{mv},x_{target}\right)=\int_{-\infty }^{\infty }L\left(x_{cursor},x_{target}\right)p\left(x_{cursor}|x_{aim},\sigma_{mv}\right)dx_{cursor}.$$ (10)

Finally, the statistically optimal location to aim the hand that minimizes squared error [$x_{aim}^{min(error^{2})}$] is found by,

$$x_{aim}^{min(error^{2})}=-\underset{x_{aim}}{arg min}[EL(x_{aim},\sigma_{mv},x_{target})].$$ (11)

The negative sign in front of this equation accounts for $x_{aim}^{min(error^{2})}$ being a compensation equal to and in the opposite direction of the average shift in cursor position caused by the skewed lateral shift probability distribution. Further, it should be noted that $x_{aim}^{min(error^{2})}$ is invariant to differences in movement variability and corresponds to the mean of *p*(*x*_*cursor*_|*x*_*aim*_, *σ*_*mv*_). Thus, for each group, $x_{aim}^{min(error^{2})}$ is equal to 5.4*mm*. Please refer to Fig 4B for a visual example of where one would aim their hand to minimize the sum of squared cursor errors around the displayed target.

We also calculated the optimal location to aim the hand that maximizes the probability of hitting the target $\left[x_{aim}^{max(hits)}\right]$. To do this, we found the location to aim the hand that placed the mode of *p*(*x*_*cursor*_|*x*_*aim*_, *σ*_*mv*_) directly over the displayed target (Fig 4C) by minimizing their distance from one another. This is equivalent to minimizing the 0-1 loss function and can be summarized as

$$x_{aim}^{max(hits)}=-\underset{x_{aim}}{arg min}\{|\underset{x_{cursor}}{arg max}[p(x_{cursor}|x_{aim},\sigma_{mv})]-x_{target}|\},$$ (12)

where the mode location of *p*(*x*_*cursor*_|*x*_*aim*_, *σ*_*mv*_) was found numerically using an arg max function. We found the estimated values of $x_{aim}^{max(hits)}$ for each group: *Reinforcement* = 10.7*mm*, *Error* = 11.5*mm*, and *Reinforcement* + *Error* = 11.7*mm*. Slight differences between groups occur because the mode location of *p*(*x*_*cursor*_|*x*_*aim*_, *σ*_*mv*_) is influenced by movement variability (unlike Eq 11). The negative sign in the front of this equation represents a compensation to the additive effects of movement variability and the skewed lateral shift probability distribution on cursor position.

For the three experimental groups, compensation reached the average asymptotic level by trial 100 (Fig 6A). Thus, for each participant, we averaged their last 400 trials to obtain a stable estimate of their adapted behavior (Fig 6B). The results were robust to whether we averaged the last 100, 200, 300 or 400 trials.

Based on the result of Experiment 1, we expected both the *Error* and *Reinforcement* + *Error* groups in Experiment 2 to minimize approximately squared error and compensate for the imposed lateral shifts by an amount that corresponds closely to the mean of the skewed lateral shift probability distribution. Thus, we expected no significant differences in compensation between these groups.

We expected that the *Reinforcement* group would aim towards $x_{aim}^{max(hits)}$. We hypothesized that their average aim location would be significantly different from both the *Error* and *Reinforcement* + *Error* groups.

If the patterns of compensation associated with the *Reinforcement* group and *Error* group were significantly different, this would support the idea that the sensorimotor system can use at least two dissociable loss functions to update where to aim the hand during a reach—one that minimizes error and another that maximizes the probability of hitting the target.

Further, based on the results of Experiment 1, we expected the *Reinforcement* + *Error* group would compensate in the same manner as the *Error* group but differently from the *Reinforcement* group. This would support the idea that, providing the behavior of the *Error* and *Reinforcement* groups was dissociable, the sensorimotor system heavily weights error feedback over reinforcement.

### Statistical analysis

SPSS (version 21.0; IBM, Armond, NY) was used for analysis of variance (ANOVA) tests. We used Greenhouse-Geisser adjustments to correct for violations of sphericity. Effect sizes for each ANOVA were calculated using generalized omega squared (${\hat{\omega }}_{G}^{2}$), where values of ${\hat{\omega }}_{G}^{2}$ equal to 0.02, 0.13, and 0.26 were considered small, medium and large effects [56, 57]. Follow-up comparisons (one-sampled, two-sampled or paired) were computed using nonparametric bootstrap hypothesis tests (*n* = 1,000,000). These tests provide more reliable *p*-value estimates than traditional comparison tests (e.g., t-tests). Briefly, they make no parameter assumptions (e.g., normality) and are less sensitive to unequal sample sizes or unequal variances. One-tailed tests were used when we had a priori predictions based on our Bayesian model, such as when we predicted significantly different compensation between participants only receiving error feedback and those receiving only reinforcement feedback. For all other comparisons we used two-tailed tests. Holm-Bonferroni corrections were used to allow for multiple comparisons [58]. 95^th^ percentile confidence intervals (*CI*) were calculated by bootstrapping. Effect sizes for follow-up comparisons were made using the common language effect size statistic ($\hat{\theta }$), where values of $\hat{\theta }$ equal to 56.0%, 64.0% and 71.0% were respectively considered small, medium and large effects [59, 60]. Significance was set to *p* < 0.05.

#### Experiment 1

A one-way ANOVA, with group as the independent variable (*Error*_*SR*_, *Error*_*SL*_, *Reinforcement* + *Error*_*SR*_), was used to assess the average compensation when visual error feedback was withheld. We also performed a one-way ANOVA on the dependent variable *α*^*opt*^ with group (*Error*_*SR*_, *Error*_*SL*_, *Reinforcement* + *Error*_*SR*_) as the independent variable. Bootstrap tests were used to compare *α*^*opt*^ values to 1.0 and 2.0, which corresponded to minimizing absolute and squared error, respectively. We also compared *α*^*opt*^ to 1.72 (±0.008*SE*), which was the power-loss function exponent reported by Körding and Wolpert (2004b).

#### Experiment 2

We performed a two-way, factorial ANOVA on participants’ average compensation over the last 400 trials with group (*Reinforcement*, *Error*, *Reinforcement* + *Error*) and skew direction (*SR*, *SL*) as the independent variables. For participants who only received reinforcement feedback, we calculated the coefficient of determination (*R*^2^) between their movement variability and final compensation. Bootstrap tests were used to compare the compensation of each group to their calculated $x_{aim}^{max(hits)}$ and $x_{aim}^{min(error^{2})}$.

## Supporting information

S1 Data Bayesian model fitting and optimal parameters. An example of an individual’s data and corresponding Bayesian model fit. Average best-fit parameters of the Bayesian model for each group. Statistical tests demonstrating the nervous system is integrating prior and current information in a statistically optimal way.
(PDF)

S2 Data Linear model fitting. The best-fit parameters to individual data. Statistical tests of the linear model: a) further support the finding that reaching behavior was not influenced by reinforcement feedback in Experiment 1 and b) show that the nervous system is integrating prior and current information in a statistically optimal way.
(PDF)

## References

[1] Izawa J, Shadmehr R (2011) Learning from sensory and reward prediction errors during motor adaptation. PLoS Comput Biol 7(3): e1002012 10.1371/journal.pcbi.1002012 21423711 PMC3053313

[2] Haith AM, Krakauer JW (2013) Model-based and model-free mechanisms of human motor learning In Progress in motor control (pp. 1–21). Springer New York. 10.1007/978-1-4614-5465-6_1 PMC3570165 23296478

[3] Shmuelof L, Huang VS, Haith AM, Delnicki RJ, Mazzoni P, Krakauer JW (2012) Overcoming motor “forgetting” through reinforcement of learned actions. J Neurosci 23(42): 14617–14621. 10.1523/JNEUROSCI.2184-12.2012 PMC3525880 23077047

[4] Wolpert DM, Ghahramani Z, Jordan MI (1995) An internal model for sensorimotor integration. Science 269(5232): 1880 10.1126/science.7569931 7569931

[5] Huang VS, Haith A, Mazzoni P, Krakauer JW (2011) Rethinking motor learning and savings in adaptation paradigms: model-free memory for successful actions combines with internal models. Neuron 70(4): 787–801. 10.1016/j.neuron.2011.04.012 21609832 PMC3134523

[6] Faisal AA, Selen LP, Wolpert DM (2008) Noise in the nervous system. Nat Rev Neurosci 9(4): 292–303. 10.1038/nrn2258 18319728 PMC2631351

[7] Trommershäuser J, Maloney LT, and Landy MS (2003a) Statistical decision theory and trade-offs in the control of motor response. Spat Vis 16: 255–275. 10.1163/156856803322467527 12858951

[8] Körding KP, Wolpert DM (2004a) Bayesian integration in sensorimotor learning. Nature 427(6971): 244–247. 10.1038/nature02169 14724638

[9] Körding KP, Wolpert DM (2004b) The loss function of sensorimotor learning. PNAS 101(26): 9839–9842. 10.1073/pnas.0308394101 15210973 PMC470761

[10] Wolpert DM, Landy MS (2012) Motor control is decision-making. Curr Opin Neurol 22(6): 996–1003. 10.1016/j.conb.2012.05.003 PMC3434279 22647641

[11] Cashaback JGA, McGregor HR, Pun H, Buckingham G, Gribble PL (2017). Does the sensorimotor system minimize prediction error or select the most likely prediction during object lifting? J Neurophysiol 117(1): 260–274. 10.1152/jn.00609.2016 27760821 PMC5220115

[12] Vaswani PA, Shmuelof L, Haith AM, Delnicki RJ, Huang VS, Mazzoni P, Shadmehr R, Krakauer JW (2015) Persistent residual errors in motor adaptation tasks: reversion to baseline and exploratory escape. J Neurosci 35(17): 6969–6977. 10.1523/JNEUROSCI.2656-14.2015 25926471 PMC4412906

[13] Sensinger J, Aleman-Zapata A, Englehart K (2015) Do Cost Functions for Tracking Error Generalize across Tasks with Different Noise Levels? PloS One 10(8): e0136251 10.1371/journal.pone.0136251 26313560 PMC4552421

[14] Scheidt RA, Dingwell JB, Mussa-Ivaldi FA (2001) Learning to move amid uncertainty. J Neurophysiol 86(2): 971–985. 11495965 10.1152/jn.2001.86.2.971

[15] Zhang H, Daw ND, Maloney LT (2015) Human representation of visuo-motor uncertainty as mixtures of orthogonal basis distributions. Nat Neurosci 18(8): 1152–1158. 10.1038/nn.4055 26120962 PMC4487408

[16] Acerbi L, Vijayakumar S, Wolpert DM (2014) On the origins of suboptimality in human probabilistic inference. PLoS Comput Biol 10(6): e1003661 10.1371/journal.pcbi.1003661 24945142 PMC4063671

[17] Nikooyan AA, Ahmed AA (2015) Reward feedback accelerates motor learning. J Neurophysiol 113(2): 633–646. 10.1152/jn.00032.2014 25355957

[18] Pekny SE, Izawa J, Shadmehr R (2015) Reward-dependent modulation of movement variability. J Neurosci 35(9): 4015–4024. 10.1523/JNEUROSCI.3244-14.2015 25740529 PMC4348194

[19] Galea JM, Mallia E, Rothwell J, Diedrichsen J (2015) The dissociable effects of punishment and reward on motor learning. Nat Neurosci 18(4): 597–602. 10.1038/nn.3956 25706473

[20] Trommershäuser J, Maloney LT, Landy MS (2003b) Statistical decision theory and the selection of rapid, goal-directed movements. JOSA A 20(7): 1419–1433. 10.1364/JOSAA.20.001419 12868646

[21] Trommershäuser J, Gepshtein S, Maloney LT, Landy MS, Banks MS (2005) Optimal compensation for changes in task-relevant movement variability. J Neurosci 25(31): 7169–7178. 10.1523/JNEUROSCI.1906-05.2005 16079399 PMC6725228

[22] Trommershäuser J, Landy MS, Maloney LT (2006a) Humans rapidly estimate expected gain in movement planning. Psychol Sci 17(11): 981–988. 10.1111/j.1467-9280.2006.01816.x 17176431

[23] Trommershäuser J, Mattis J, Maloney LT, Landy MS (2006b) Limits to human movement planning with delayed and unpredictable onset of needed information. Exp Brain Res 175(2): 276–284. 10.1007/s00221-006-0546-z 16736179

[24] Mazzoni P, Krakauer JW (2006) An implicit plan overrides an explicit strategy during visuomotor adaptation. J Neurosci 26: 3642–3645. 10.1523/JNEUROSCI.5317-05.2006 16597717 PMC6674132

[25] Chalk M, Seitz A, Series P (2010) Rapidly learned stimulus expectations alter perception of motion. J Vis 10: 1–18. 10.1167/10.8.2 20884577

[26] Marko MK, Haith AM, Harran MD, Shadmehr R (2012) Sensitivity to prediction error in reach adaptation. J Neurophysiol 108: 1752–1763. 10.1152/jn.00177.2012 22773782 PMC3774589

[27] Trampenau L, Kuhtz-Buschbeck JP, van Eimeren T (2015) Probabilistic information on object weight shapes force dynamics in a grip-lift task. Exp Brain Res 233: 1711–1720. 10.1007/s00221-015-4244-6 25761969

[28] Körding KP, Wolpert DM (2003). Probabilistic Inference in Human Sensorimotor Processing. NIPS: 1327–1334.

[29] Peters MA, Wei JM, Shams L (2016) The size-weight illusion in not anti-Bayesian after all: a unifying Bayesian account. PeerJ 4: e2124 10.7717/peerj.2124 27350899 PMC4918219

[30] Crevecoeur F, Kording KP (2017). Saccadic suppression as a perceptual consequence of efficient sensorimotor estimation. eLife 6: e25073 10.7554/eLife.25073 28463113 PMC5449188

[31] Thoroughman KA, Shadmehr R (2000) Learning of action through adaptive combination of motor primitives. Nature 407: 742–747. 10.1038/35037588 11048720 PMC2556237

[32] Baddeley R.J., Ingram H.A., and Miall R.C. (2003). System identification applied to a visuomotor task: near-optimal human performance in a noisy changing task. J. Neurosci. 23, 3066–3075. 12684493 10.1523/JNEUROSCI.23-07-03066.2003 PMC6742112

[33] Diedrichsen J, Hashambhoy Y, Rane T, Shadmehr R (2005) Neural correlates of reach errors. J Neurosci 25: 9919–9931. 10.1523/JNEUROSCI.1874-05.2005 16251440 PMC1479774

[34] Smith MA, Ghazizadeh A, Shadmehr R (2006) Interacting adaptive processes with different timescales underlie short-term motor learning. PLoS Biol, 4(6), e179 10.1371/journal.pbio.0040179 16700627 PMC1463025

[35] Burge J, Ernst MO, Banks MS (2008) The statistical determinants of adaptation rate in human reaching. J Vis 8: 1–19. 10.1167/8.4.20 18484859 PMC2684526

[36] van Beers RJ (2009) Motor learning is optimally tuned to the properties of motor noise. Neuron 63(3): 406–417. 10.1016/j.neuron.2009.06.025 19679079

[37] van Beers RJ, van der Meer Y, Veerman RM (2013a) What autocorrelation tells us about motor variability: insights from dart throwing. PloS one 8(5): e64332 10.1371/journal.pone.0064332 23691199 PMC3656833

[38] van Beers RJ, Brenner E, Smeets JB (2013b) Random walk of motor planning in task-irrelevant dimensions. J Neurophysiol 109(4): 969–977. 10.1152/jn.00706.2012 23175799

[39] Gepshtein S, Seydell A, Trommershäuser J (2007) Optimality of human movement under natural variations of visual-motor uncertainty. J Vision 7(5): 13–13. 10.1167/7.5.13 18217853

[40] Stritzke M, Trommershäuser J (2007) Eye movements during rapid pointing under risk. Vision Res 47(15): 2000–2009. 10.1016/j.visres.2007.04.013 17532361

[41] Taylor JA, Krakauer JW, Ivry RB (2014) Explicit and implicit contributions to learning in a sensorimotor adaptation task. J Neurosci 34(8): 3023–3032. 10.1523/JNEUROSCI.3619-13.2014 24553942 PMC3931506

[42] Wu HG, Miyamoto YR, Castro LNG, Ölveczky BP, Smith MA (2014) Temporal structure of motor variability is dynamically regulated and predicts motor learning ability. Nat Neurosci 17(2): 312–321. 10.1038/nn.3616 24413700 PMC4442489

[43] Cashaback JGA, Pierrynowski MR, Potvin JR (2013a) Calculating individual and total muscular translational stiffness: a knee example. J Biomech Eng-T ASME 135(6): 061006 10.1115/1.4024162 23699718

[44] Cashaback JGA, Potvin JR, Pierrynowski MR (2013b) On the derivation of a tensor to calculate six degree-of-freedom, musculotendon joint stiffness: Implications for stability and impedance analyses. J Biomech 46(15): 2741–2744. 10.1016/j.jbiomech.2013.07.020 24028892

[45] Cashaback JGA, Cluff T, Potvin JR (2013c) Muscle fatigue and contraction intensity modulates the complexity of surface electromyography. J Electromyogr Kines 23(1): 78–83. 10.1016/j.jelekin.2012.08.004 22959820

[46] Harris CM, Wolpert DM (1998) Signal-dependent noise determines motor planning. Nature 394(6695): 780–784. 10.1038/29528 9723616

[47] Buzsáki G, Mizuseki K (2014). The log-dynamic brain: how skewed distributions affect network operations. Nat Rev Neurosci 15(4): 264–278. 10.1038/nrn3687 24569488 PMC4051294

[48] Box GE, Muller ME (1958) A note on the generation of random normal deviates. Ann Math Stat 29(2): 610–611. 10.1214/aoms/1177706645

[49] Wong JD, Wilson ET, Gribble PL (2011) Spatially selective enhancement of proprioceptive acuity following motor learning. J Neurophysiol 105(5): 2512–2521. 10.1152/jn.00949.2010 21368000 PMC3094168

[50] Bernardi NF, Darainy M, Ostry DJ (2015) Somatosensory contribution to the initial stages of human motor learning. J Neurosci 35(42): 14316–14326. 10.1523/JNEUROSCI.1344-15.2015 26490869 PMC4683690

[51] Nelder JA, Mead R (1965) A simplex method for function minimization. Comput J 7(4): 308–313. 10.1093/comjnl/7.4.308

[52] Bassett G Jr, Koenker R (1978) Asymptotic theory of least absolute error regression. JASA 73(363): 618–622. 10.1080/01621459.1978.10480065

[53] Landy MS, Trommershäuser J, Daw ND (2012) Dynamic estimation of task-relevant variance in movement under risk. J Neurosci 32(37): 12702–12711. 10.1523/JNEUROSCI.6160-11.2012 22972994 PMC3477850

[54] Cashaback JGA, McGregor HR, Gribble PL (2015) The human motor system alters its reaching movement plan for task-irrelevant, positional forces. J Neurophysiol 113(7): 2137–2149. 10.1152/jn.00901.2014 25589594 PMC4416597

[55] Herzfeld D. J., Shadmehr R. (2014). Motor variability is not noise, but grist for the learning mill. Nat Neurosci 17(2), 149–150. 10.1038/nn.3633 24473260

[56] Olejnik S, Algina J (2003) Generalized eta and omega squared statistics: measures of effect size for some common research designs. Psychol Methods 8(4): 434–447. 10.1037/1082-989X.8.4.434 14664681

[57] Bakeman (2005) Recommended effect size statistics for repeated measures designs. Behav Res Methods 37(3): 379–384. 10.3758/BF03192707 16405133

[58] Holm S (1979) A simple sequentially rejective multiple test procedure. Scand J Stat: 65–70.

[59] Cohen J (1988) Statistical power analysis for the behavioural sciences. Hillside. NJ: Lawrence Earlbaum Associates.

[60] McGraw KO, Wong SP (1992) A common language effect size statistic. Psychol Bull 111(2): 361–365. 10.1037/0033-2909.111.2.361

