# Supplementary material for: Dissociating error-based and reinforcement-based loss functions during sensorimotor learning
Source: PLoS Comput Biol. 2017 Jul 28;13(7):e1005623. doi: 10.1371/journal.pcbi.1005623 (PMC5550011; doi:10.1371/journal.pcbi.1005623)
Supplement: S1 Data — An example of an individual’s data and corresponding Bayesian model fit. Average best-fit parameters of the Bayesian model for each group. Statistical tests demonstrating the nervous system is integrating prior and current information in a statistically optimal way. (PDF) [file pcbi.1005623.s001.pdf]

BAYESIAN MODEL FITTING AND OPTIMAL PARAMETERS

Our Bayesian model describes how participants combine previously acquired information about lateral shifts (prior) with sensed visual feedback of a lateral shift (likelihood) to generate an estimate of the lateral shift (posterior). This lateral shift estimate is then used to generate a compensation for any given lateral shift on a trial by trial basis. The four fitted parameters of the Bayesian model provide an estimate of how error was minimized (α^{opt}) and participants' estimate of uncertainty (σ_1^{opt}, σ_2^{opt}, σ_3^{opt}) for the single dot, medium cloud and large cloud of dots, respectively. An example of a participant's behavior and their corresponding best-fit model is shown in **Fig. S1**.

The results of α^{opt} are discussed and displayed in the Results section of the manuscript and in this Supplementary (**Fig. 3D** and **Fig. S2**, respectively). In summary, we found that reinforcement feedback did not influence behavior when provided in combination with error feedback. This resulted in all three groups minimizing approximately squared error.

The three fitted parameters, σ_1^{opt}, σ_2^{opt}, σ_3^{opt}, represent the average participant uncertainty associated with a lateral shift, which was presented visually as either a single dot (ς_{0mm}), medium cloud of dots (ς_{15mm}), or large cloud of dots (ς_{30mm}). In the Bayesian model, larger values of σ_j^{opt} correspond to a less informative likelihood. With a less informative likelihood, we would expect participants to rely more heavily on the prior. Thus, across participants, if $\sigma_1^{opt} < \sigma_2^{opt} < \sigma_3^{opt}$, this would support the idea that the sensorimotor system accounts for environmental uncertainty in a way that aligns with Bayesian inference (see **Methods**).

A two-way, mixed factorial ANOVA was used to assess our estimate of participants' visual uncertainty (σ_j^{opt}). Here, imposed visual uncertainty (ς_{0mm}, ς_{15mm}, ς_{30mm}) and group ($Error_{SR}$, $Error_{SL}$, $Reinforcement + Error_{SR}$) were independent variables. To test whether participants were combining prior experience and current information about lateral shifts in a statistically optimal way, we examined the effect of imposed visual uncertainty (ς_j) on model estimates of participants' visual uncertainty σ_j^{opt}. As expected, we found a significant main effect of imposed visual uncertainty on model estimates of participants' visual uncertainty (σ_j) [$F(1.2, 31.2) = 37.778$,

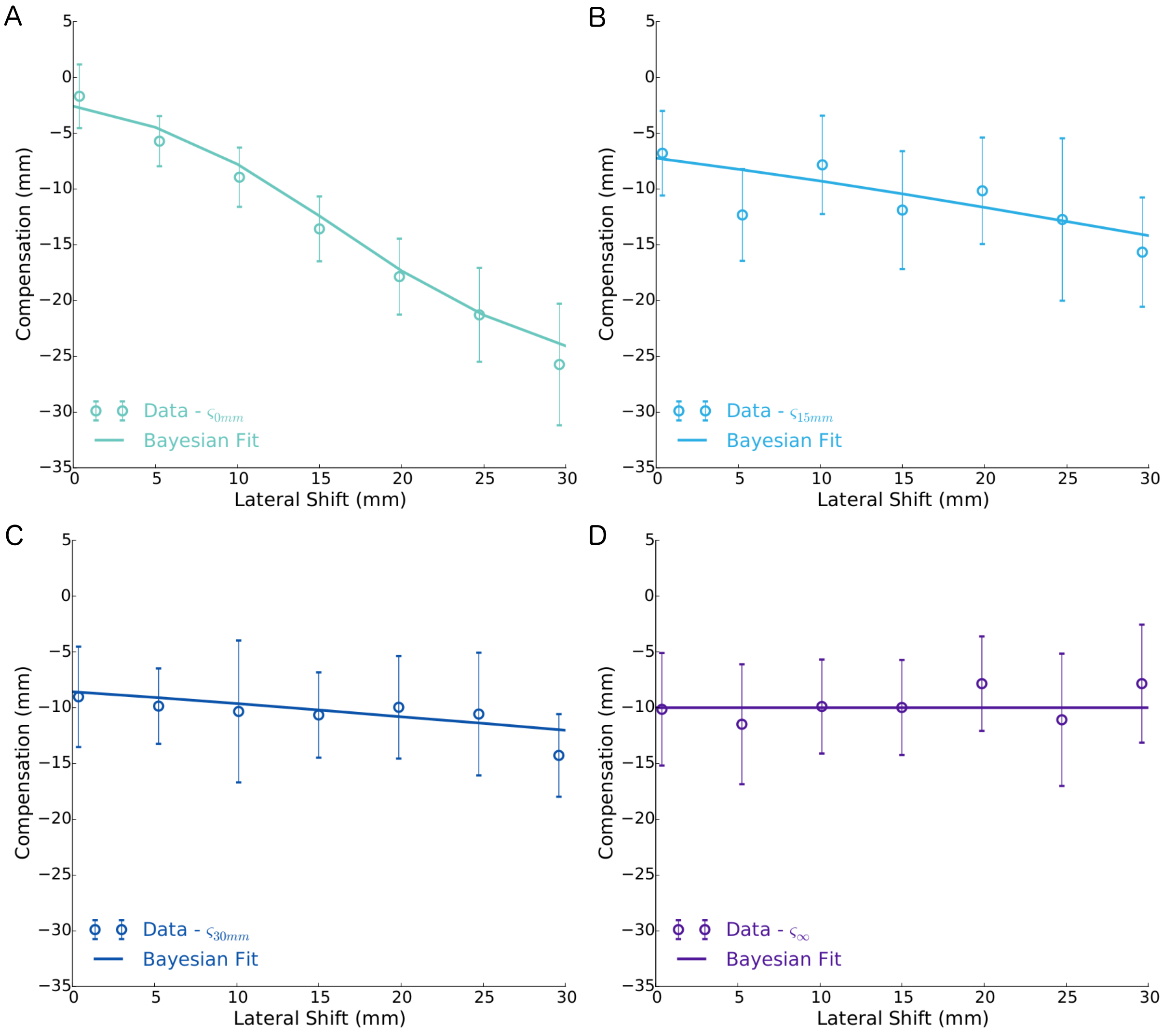

Figure S1: An individual's pattern of compensation (unfilled circles) and corresponding Bayesian fit (solid line) for different magnitudes of lateral shift (x-axes). Each panel corresponds to whether visual (error) feedback was provided as **A)** a single dot (ς_{0mm}), **B)** a medium cloud of dots (ς_{15mm}), **C)** a large cloud of dots (ς_{30mm}) or **D)** withheld (ς_{∞}). Error bars represent $\pm$ 1 standard deviation.

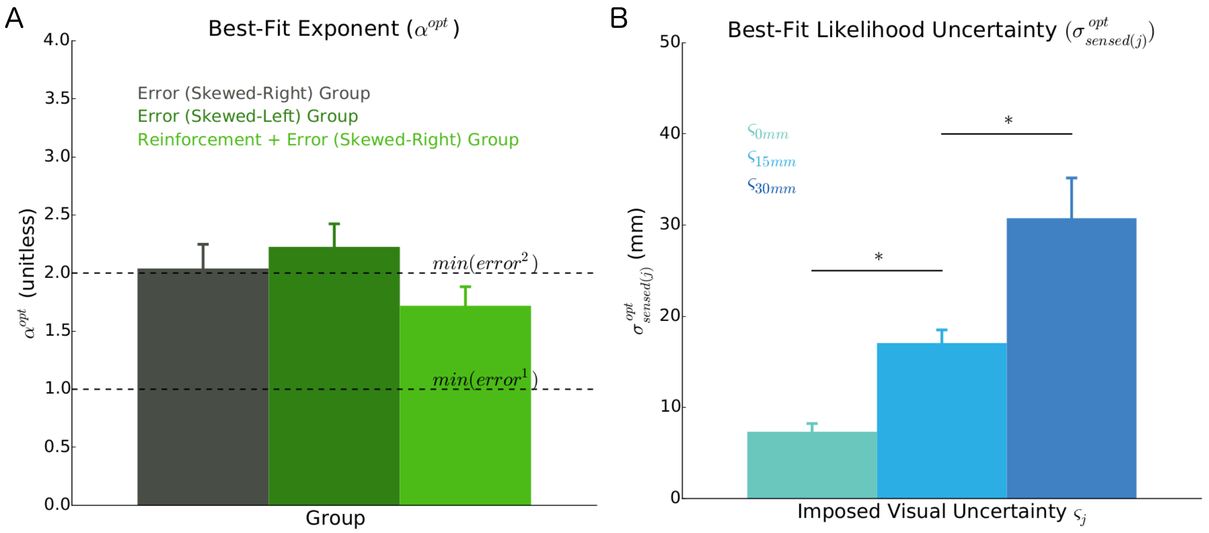

Figure S2: Best-fit, Bayesian model parameters. **A)** The average power-loss function exponent (α^{opt}) of each group. There were no significant differences between groups. An exponent of 2.0 corresponds to minimizing squared error (upper dashed line), while an exponent of 1.0 corresponds to minimizing absolute error (lower dashed line). **B)** Estimates of participant visual uncertainty (α^{opt}, σ_1^{opt}, σ_2^{opt}, σ_3^{opt}) for different amounts of imposed visual uncertainty (single dot = ς_{0mm}; medium cloud of dots = ς_{15mm}; large cloud of dots = ς_{30mm}, respectively). Error bars represent ± 1 standard error of the mean. $*p < 0.05$.

$p < 0.001$, $\hat{\omega}_G^2 = 0.245$]. There was no effect of group [$F(2, 27) = 3.132$, $p = 0.060$, $\hat{\omega}_G^2 = 0.042$] nor an interaction between group and imposed uncertainty [$F(2.3, 31.2) = 1.157$, $p = 0.333$, $\hat{\omega}_G^2 = 0.002$]. Planned, paired bootstrap tests showed, with a change in imposed visual uncertainty (ς_j), that $\sigma_1^{opt} << \sigma_2^{opt} << \sigma_3^{opt}$, where p < 0.001 and $\hat{\theta} \geq 90.0\%$ was found for each comparison (**Fig. S2B**). These data are consistent with the idea that, with decreases in error feedback quality, participants were performing in a way aligned with Bayesian inference by systematically relying more on previous experience (i.e., the prior) and less on mid-reach trial by trial feedback.

For each group, a coefficient of determination (R^2) was computed to examine the quality of fit between participants' compensatory behavior ($comp_{i,j}^{data}$) and our estimates of optimal positional compensation ($comp_{i,j}^{opt}$) based on the best-fit parameters (α^{opt}, σ_1^{opt}, σ_2^{opt}, σ_3^{opt}). To further assess the ability of our Bayesian model to describe behavior, we compared participants' compensatory pattern of compensation ($comp_{i,j}^{data}$) to predictions made from our Bayesian model ($comp_{i,j}^{opt}$). As seen in **Fig. S3**, we found a strong relationship between behavioural measures and

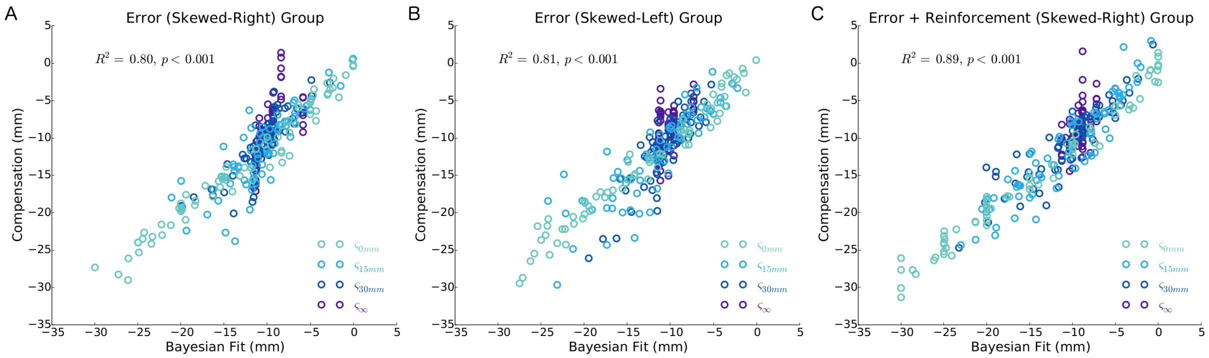

Figure S3: Comparison of individual patterns of compensation (unfilled circles) to the outputs of their best-fit Bayesian model are shown, for all 28 combinations of lateral shift and imposed visually uncertainty (ς_j), for each participant receiving **A)** error feedback laterally shifted by skewed-right probability distribution distribution, **B)** error feedback laterally shifted by skewed-left probability distribution, and **C)** both reinforcement and error feedback laterally shifted by skewed-right probability distribution.

model predictions for each group. Specifically, for the $Error_{SR}$, $Error_{SL}$, $Reinforcement + Error_{SR}$, we found significant correlations between $comp_{i,j}^{data}$ and $comp_{i,j}^{opt}$; R^2 values were 0.80 ($p \leq 0.001$), 0.81 ($p \leq 0.001$) and 0.89 ($p \leq 0.001$), respectively.
