# Supplementary material for: Dissociating error-based and reinforcement-based loss functions during sensorimotor learning
Source: PLoS Comput Biol. 2017 Jul 28;13(7):e1005623. doi: 10.1371/journal.pcbi.1005623 (PMC5550011; doi:10.1371/journal.pcbi.1005623)
Supplement: S2 Data — The best-fit parameters to individual data. Statistical tests of the linear model: a) further support the finding that reaching behavior was not influenced by reinforcement feedback in Experiment 1 and b) show that the nervous system is integrating prior and current information in a statistically optimal way. (PDF) [file pcbi.1005623.s002.pdf]

LINEAR MODEL FITTING

In addition to the Bayesian model, we also fit the behavioural data from Experiment 1 using a simple linear model. Importantly, the linear model (details below) yielded the same findings as the Bayesian model. That is, there were no significant differences between the three experimental groups ($Error_{SR}$, $Error_{SL}$, $Reinforcement{+}Error_{SR}$), and a strong effect of imposed visually uncertainty (ς_{0mm}, ς_{15mm}, ς_{30mm}, ς_{∞}) on behaviour. These results further support our finding that reinforcement feedback did not play a role in influencing behaviour when combined with error feedback, and that the sensorimotor system responds to different amounts of visual uncertainty.

Two advantages of this linear model are: 1) it allows us to demonstrate that the results of Experiment 1 are robust and generalizable across different classes of models, and 2) it is more agnostic in describing the observed behaviour as it makes fewer assumptions on the underlying mechanisms (e.g., Bayesian integration) that the sensorimotor system may be employing to direct the aim location of the hand during reaching. It is, however, important to note that the Bayesian model used within the manuscript aligns with current putative theories of sensorimotor behaviour and explained between 80% to 90% of the observed variance using only four free parameters, as opposed to the eight free parameters of the linear model.

Using the linear model, for each participant we found the line of best fit between the true lateral shift and their corresponding compensation given the amount of imposed visual uncertainty (ς_{0mm}, ς_{15mm}, ς_{30mm}, ς_{∞}). Thus, there were a total of 8 parameters (4 slopes and 4 intercepts: 1 each per condition) used to fit the data of each participant. **Fig. S1** shows the relationship between the slopes and intercepts from the linear model that was fit to each participant.

As shown by others, we expected both the $Error_{SR}$, $Error_{SL}$ groups to minimize approximately squared error (Scheidt et al., 2001; Zhang et al., 2015; Cashaback et al., 2017). Thus, with decreases in visual certainty, we expected the intercepts of the groups receiving only error feedback to approach a value that corresponds to the mean of the skewed lateral shift probability distribution.

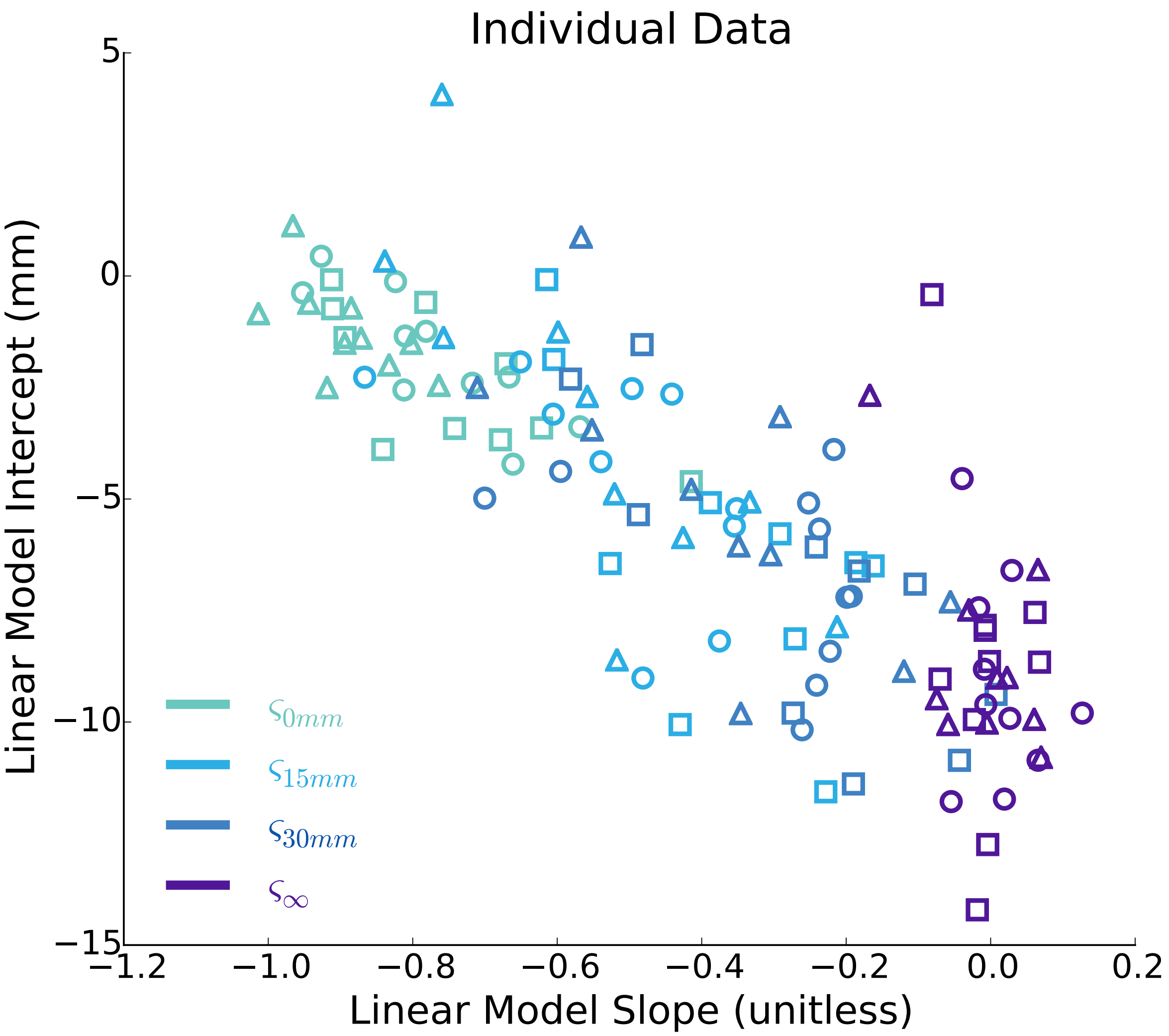

Figure S1: Scatterplot of the slopes (x-axis) and intercepts (y-axis) from the linear model that was fit to each participant (n = 30) and the four conditions of visual uncertainty (the darker the shade the greater the visual uncertainty). The square, circle and triangle markers correspond to individuals in the $Error_{SR}$, $Error_{SL}$, $Reinforcement{+}Error_{SR}$ groups, respectively. Visual inspection and statistical tests reveal no differences between groups. In the no vision condition (ς_{∞}: darkest blue), all three groups have an intercept that is clustered around $-10.0mm$—demonstrating that participants minimized approximately squared error. This indicates that participants were compensating to the lateral shifts by an amount corresponding to the mean of the skewed lateral shift probability distribution as opposed to a compensation aligned with the mode ($0.0mm$). Across participants it can also be seen that the different levels of imposed uncertainty resulted in significantly different linear model slopes. This suggests that the sensorimotor system integrates uncertain visual information in a probabilistically optimal way.

Our initial hypothesis postulated that humans increasingly rely on reinforcement feedback with a decrease in error feedback quality. Based on this hypothesis, from the linear model we would expect that the intercepts of the $Reinforcement + Error_{SR}$ to be significantly different from the groups receiving only error feedback ($Error_{SR}$, $Error_{SL}$). Specifically, with decreases in visual certainty, we expected the $Reinforcement + Error_{SR}$ participants to have their intercepts move away from an amount corresponding to the mean of the skewed lateral shift probability distribution and approach an amount corresponding to the mode. As shown by Körding and Wolpert (2004a), another expectation was that the nervous system would compensate for the different magnitudes of the imposed visual uncertainty in probabilistically optimal way. For the linear model, this would manifest itself as significantly different slopes (and consequently intercepts) as a function of imposed visual uncertainty.

We assessed both the intercepts and slopes of the linear model using separate two-way, 3 (group: $Error_{SR}$, $Error_{SL}$, $Reinforcement + Error_{SR}$) x 4 (imposed visual uncertainty: ς_{0mm}, ς_{15mm}, ς_{30mm}, ς_{∞}) mixed analysis of variance. Follow-up comparisons were computed using non-parametric bootstrap hypothesis tests. General omega squared ($\hat{\omega}^2_G$) and common language effect size ($\hat{\theta}$) statistics were used to characterize the effect size of omnibus and comparison tests, respectively.

As with our Bayesian model, we found no significant main effect of group [$F(2, 27) = 1.111$, $p = 0.344$, $\hat{\omega}^2_G = 0.003$] nor a significant interaction of group and imposed visual uncertainty [$F(4.3, 57.9) = 1.296$, $p = 0.281$, $\hat{\omega}^2_G = 0.005$] for the intercepts of the linear model. These finding contradicts our initial hypothesis, where we expected that participants who received both error and reinforcement feedback would shift their behavior from minimizing error to maximizing the probability of success. Further, there was no main effect of group [$F(2, 27) = 1.773$, $p = 0.114$, $\hat{\omega}^2_G = 0.028$] nor an interaction between group and imposed visual uncertainty [$F(3.7, 49.4) = 1.773$, $p = 0.154$, $\hat{\omega}^2_G = 0.016$] for the slopes of the linear model.

As expected, we did find a significant main effect of imposed visual uncertainty for both slopes [$F(1.8, 49.4) = 281.7$, $p < 0.001$, $\hat{\omega}_G^2$ = 0.795] and intercepts [$F(2.1, 57.9) = 87.4$, $p < 0.001$, $\hat{\omega}_G^2 = 0.459$], demonstrating that the sensorimotor system was sensitive to the imposed visual uncertainty and responded in a way that aligns with Bayesian statistics. For both dependent measures, planned paired comparisons showed that ς_{0mm}, ς_{15mm}, ς_{30mm}, ς_{∞} were sequentially significantly different from one another, where $p < 0.001$ and $\hat{\theta} \geq 83.3\%$ for all of the comparison.
